# Characterization of the Differential Response of Endothelial Cells Exposed to Normal and Elevated Laminar Shear Stress

**DOI:** 10.1002/jcp.22629

**Published:** 2011-02-01

**Authors:** Stephen J White, Elaine M Hayes, Stéphanie Lehoux, Jamie Y Jeremy, Anton JG Horrevoets, Andrew C Newby

**Affiliations:** 1 Bristol Heart Institute, University of Bristol (Clinical Sciences South Bristol)Bristol, UK; 2 Lady Davis Institute, McGill UniversityMontreal, Quebec, Canada; 3 Department of Molecular Cell Biology and Immunology, VU University Medical CenterAmsterdam, The Netherlands

## Abstract

Most acute coronary events occur in the upstream region of stenotic atherosclerotic plaques that experience laminar shear stress (LSS) elevated above normal physiological levels. Many studies have described the atheroprotective effect on endothelial behavior of normal physiological LSS (approximately 15 dynes/cm^2^) compared to static or oscillatory shear stress (OSS), but it is unknown whether the levels of elevated shear stress imposed by a stenotic plaque would preserve, enhance or reverse this effect. Therefore we used transcriptomics and related functional analyses to compare human endothelial cells exposed to laminar shear stress of 15 (LSS15-normal) or 75 dynes/cm^2^ (LSS75-elevated). LSS75 upregulated expression of 145 and downregulated expression of 158 genes more than twofold relative to LSS15. Modulation of the metallothioneins (MT1-G, -M, -X) and NADPH oxidase subunits (NOX2, NOX4, NOX5, and p67phox) accompanied suppression of reactive oxygen species production at LSS75. Shear induced changes in dual specificity phosphatases (DUSPs 1, 5, 8, and 16 increasing and DUSPs 6 and 23 decreasing) were observed as well as reduced ERK1/2 but increased p38 MAP kinase phosphorylation. Amongst vasoactive substances, endothelin-1 expression decreased whereas vasoactive intestinal peptide (VIP) and prostacyclin expression increased, despite which intracellular cAMP levels were reduced. Promoter analysis by rVISTA identified a significant over representation of ATF and Nrf2 transcription factor binding sites in genes upregulated by LSS75 compared to LSS15. In summary, LSS75 induced a specific change in behavior, modifying gene expression, reducing ROS levels, altering MAP kinase signaling and reducing cAMP levels, opening multiple avenues for future study. J. Cell. Physiol. 226: 2841–2848, 2011. © 2011 Wiley-Liss, Inc.

Shear stress is a key regulator of endothelial function that underlies protection or predilection for atherogenesis in the underlying intima at different arterial sites (Dai et al., 2004). Compared to oscillatory shear stress (OSS) or the artifactual static conditions of cell culture, constant pulsatile laminar shear stress (LSS) within the normal physiological range induces a “quiescent” endothelial phenotype, resistant to inflammatory stimuli (Mukai et al., 2007; Partridge et al., 2007). LSS decreases NADPH oxidase activity with a concomitant reduction in reactive oxygen species (ROS), increasing the bioavailability of nitric oxide (NO) and protecting endothelial cells from apoptosis (Mohan et al., 1997, 1999; Dai et al., 2004; Duerrschmidt et al., 2006). Integrin-mediated signaling linked to selective MAP kinase activation has also been implicated in transducing the protective effects of LSS (Lehoux, 2006). The protective/quiescent phenotype is mediated in part by the upregulation and action of the transcription factors KLF-2 (Dekker et al., 2002, 2006; Lin et al., 2005; Parmar et al., 2006) and Nrf2 (Dai et al., 2007). Conversely, OSS or abrupt changes in shear stress precipitate a number of processes that activate endothelial cells and can sensitize cells to apoptosis (Mohan et al., 1997; Dekker et al., 2002; Dai et al., 2004; Duerrschmidt et al., 2006). Processes include increased NADPH oxidase activity, which causes excessive ROS production, scavenging of NO, NFκB activation and upregulation of adhesion molecule expression. This results in the activated/inflammatory endothelial cell phenotype that is associated with heightened susceptibility to atherosclerosis.

Atherosclerotic plaques that impose a luminal stenosis expose the overlying endothelium to an increase in shear stress. Given that shear stress relates to the third power of the radius (r^3^), an atherosclerotic plaque imposing a 40% stenosis will generate a ∼5-fold increase in shear at the point of maximum stenosis and a plaque imposing a 75% stenosis would result in a 64-fold increase. Interestingly, blood flow appears unaffected until the stenosis exceeds 75% (Gould et al., 1974). The time-averaged wall shear stress in human coronary arteries has been measured as approximately 16 dynes/cm^2^ by a number of studies (Stone et al., 2003; Wentzel et al., 2003; Joshi et al., 2004; Gijsen et al., 2008), with 12–15 dynes/cm^2^ being most frequently used to simulate normal arterial shear stress in vitro. In contrast, the wall shear stress overlying human plaques, has been measured at 50–>300 dynes/cm^2^, depending on the degree of stenosis (Stone et al., 2003; Gijsen et al., 2008; Torii et al., 2009; Ilegbusi and Valaski-Tuema, 2010; Leach et al., 2010; Teng et al., 2010). However very little is known about the response of endothelial cells to laminar shear stress elevated above normal physiological levels; the maximum in vitro shear stress applied to human endothelial cells was reported by the Moraweitz group (Duerrschmidt et al., 2006), who studied the effects of up to 50 dynes/cm^2^ on the production of ROS and the expression of NOX2 and p47 subunits of NADPH oxidase.

To study the effect of elevated shear on the endothelium, we used transcriptomics and related functional analyses to compare primary human umbilical endothelial cells (HUVECs) cultured at 15 dynes/cm^2^ (LSS15-approximately normal arterial shear stress) and fivefold higher—75 dynes/cm^2^ (LSS75-high shear stress) to determine if the increased shear stress modified endothelial behavior.

## Materials and Methods

### Cell culture

Pooled-donor HUVEC were bought from Promocell and cultured in endothelial cell growth medium kit (C22110-Promocell). HUVEC were placed in a parallel plate flow apparatus (Castier et al., 2009) and exposed to oscillatory flow—0 ± 5 dynes/cm^2^ (OSS), laminar shear stress—15 dynes/cm^2^ (LSS15), or 75 dynes/cm^2^ (LSS75) for 24 h. See Supplementary Figure III for information on flow apparatus and methods used.

### Gene expression analysis

Illumina HumanRef-8 array analysis was performed by ServiceXS (Leiden, Netherlands) on RNA prepared from p2 HUVECs cultured at LSS15 or LSS75 for 24 h (n = 4 from different batches of pooled donor HUVEC). Array data were extracted using Illumina's BeadStudio software. Normalization and statistical analysis were performed using scripts in R/Biocunductor (Gentleman et al., 2004). Please refer to supplementary information for full details on analysis. Microarray data have been submitted to the Gene Expression Omnibus (GEO) under accession no. GSE23289.

### Quantitative PCR

Quantitative PCR was performed using Qiagen Quantitech SYBR green PCR mastermix on cDNA prepared from HUVEC cultured for 24 h at OSS, LSS15, and LSS75. Standard curves were produced for each primer set and results calculated as copies of cDNA per ng total RNA. The primers used are listed in Supplementary Table IV.

### Western blotting

Following application of flow for 24 h, cells were lysed in SDS lysis buffer [2% SDS; 50 mM Tris pH 6.8; 10% glycerol]. Cell concentration in the lysate was quantified using PicoGreen (Invitrogen, Renfrew, UK) by mixing 100 µl of 1/1,000 cell lysate with 100 µl of 1/200 PicoGreen, both diluted in 10 mM Tris, 1 mM EDTA pH 8. Fluorescence was measured with excitation at 485 nm and emission 520 nm and compared to a standard curve from a known cell number. Cells (5000) were loaded per lane on denaturing SDS–polyacrylamide gels and blotted onto PVDF membranes. After blocking, membranes were probed with primary antibodies ATF2 (Abcam-ab47476, 1/1,000); ATF2 phospho T71/53 (Abcam-ab28812, 1/1,000); ERK1/2 (9102, Cell Signalling, Hitchin, Herts, UK, 1/1,000); ERK1/2 phospho T202/Y204 (9101, Cell Signalling, 1/1,000); c-Jun (60A8, 9165, Cell Signalling, 1/1,000); c-Jun phospho S63 (Santa Cruz-sc-7980R, 1/200); p38 (9212, Cell Signalling, 1/1,000); p38 phospho T180/Y182 (9211, Cell Signalling, 1/1,000) and detected with appropriate HRP-linked secondary antibodies.

### Cyclic AMP and eicosanoid analysis

In order to delineate signaling molecules that might be driving gene expression changes after 24 h of exposure to shear stress, cyclic AMP (cAMP) was measured using a competitive ELISA (900-066, Enzo Life Sciences, Exeter, UK) on cell lysates after 16 h of static culture or HUVEC exposed to OSS, LSS15, or LSS75. Cells were lysed in 0.1 M HCl, 0.1% Triton X-100 and results normalized to protein content. Similarly, thromboxane A2 and prostacyclin (PGI2) metabolites were measured in the conditioned media from these cells after 16 h. Thromboxane B2 (519031, Cayman via Cambridge Bioscience, Cambridge, UK) or 6-keto prostaglandin F1α (515211, Cayman) EIA kits were used according to the manufacturers' instructions. Thromboxane B2 was undetectable in the media of these cultures using this approach.

### Statistical analysis

Differences between groups were analyzed using One-way Analysis of Variance (ANOVA), using Tukey-Kramer Multiple Comparisons post hoc test.

## Results

### Transcriptome analysis

HUVEC were cultured for 24 h at a normal laminar shear stress (LSS15) or elevated shear stress (LSS75). Changes in gene expression were analyzed from four different batches of pooled-donor HUVEC using Illumina full-genome bead arrays and combined Baysian statistics and Gene Set Enrichment Analysis essentially as described (Schirmer et al., 2008). This analysis showed a significant and reproducible gene regulation by LSS75 versus LSS15 within the four individual biological replicates (Supplementary Fig. I). LSS75 modified endothelial gene expression, with the expression of 145 genes increasing and 158 genes decreasing significantly by more than twofold (Supplementary Table I) compared with LSS15.

In order to validate the results of the gene array, quantitative PCR was performed on cDNA from HUVEC cultured for 24 h at LSS15 or LSS75 with an additional group cultured under OSS. Standard curves were performed for all qPCRs and results presented as copies per ng of RNA. Of the 57 genes assayed by qPCR, approximately 47% (n = 27) demonstrated a monophasic response to laminar shear, such that positive or negative changes between OSS and LSS15 were amplified at LSS75 (Supplementary Table II). A further nine genes showed unique regulation by LSS75 with no change in mRNA expression between OSS and LSS15.

### Modulation of genes related to oxidant stress and ROS levels

**Fig. 1:**
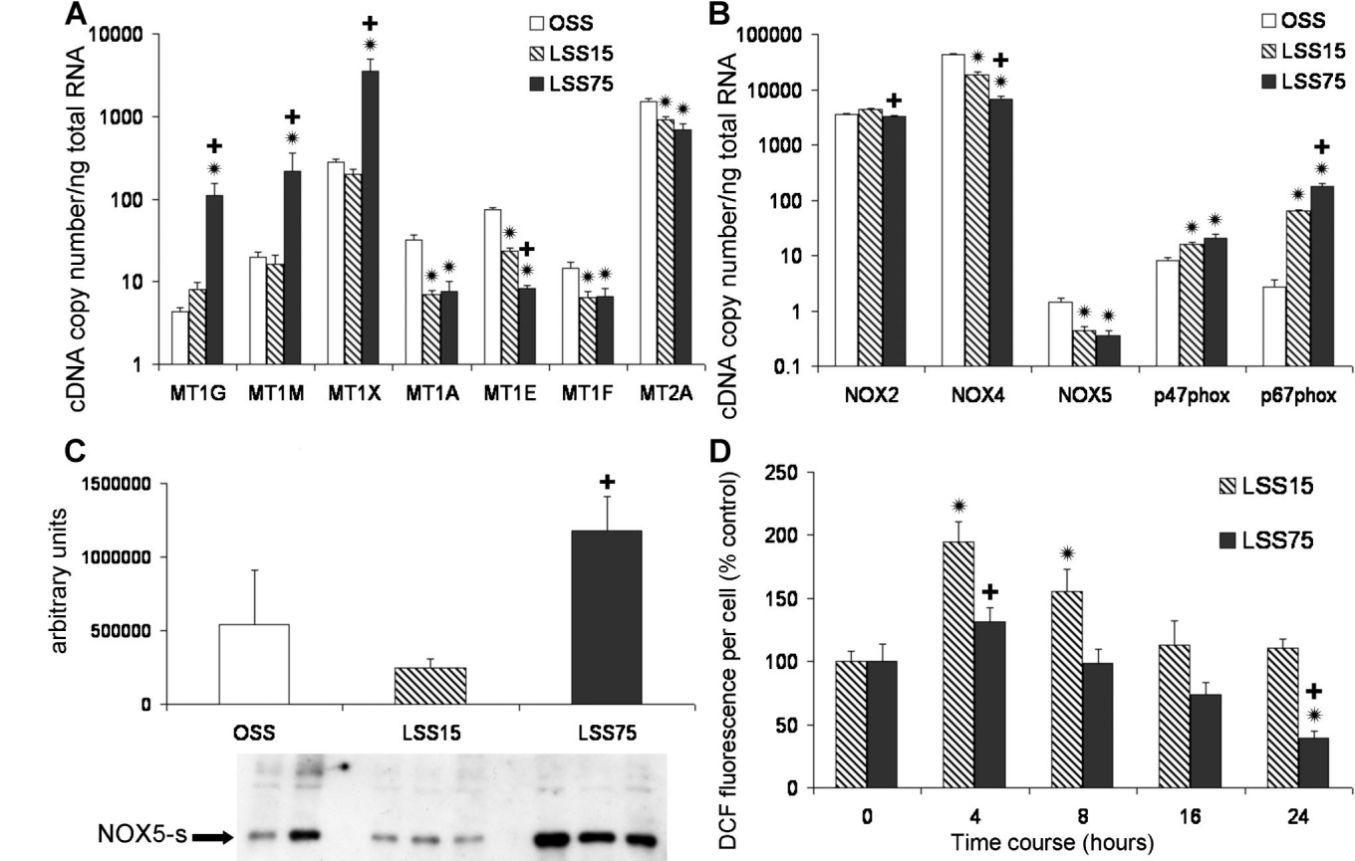
A,B: Changes in gene expression assayed by qPCR, 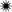 *P* < 0.05 compared to OSS; 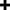 *P* < 0.05 compared to LSS15 (n = 6–9). C: Western blot assessment of NOX5 protein expression in HUVEC exposed to different shear stress regimens, 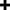 LSS75 significantly higher than LSS15 (n = 3, *P* < 0.05). D: Time course of shear stress-dependent ROS production measured by DCF fluorescence. 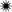 LSS75 *P* < 0.05 compared to OSS.

Among the gene families modulated by LSS75, the metallothioneins showed the biggest changes. Metallothioneins are a family of small closely related metal-binding peptides able to catalyze the dismutation of oxygen and nitrogen free radicals. They also have a signaling role having recently been demonstrated to be involved in angiogenesis and arteriogenesis (Zbinden et al., 2010). LSS75 significantly upregulated MT1G, MT1M and MT1X mRNAs approximately tenfold compared to OSS and LSS15 (Fig. 1A, note that log scales are used in a number of figures to allow comparisons between different genes to be made).

The expression of NADPH oxidase subunits were also modulated at LSS75 compared to LSS15 (Fig. 1B). LSS75 reduced the mRNA (Fig. 1B) expression of NOX2 and NOX4 compared to LSS15, but increased the expression of p67phox mRNA (Fig. 1B). Interestingly, NOX5 mRNA was reduced by exposure to LSS15 and LSS75, but the short constitutively active form was enhanced at the protein level by LSS75 compared to LSS15 (Fig. 1C). These gene expression changes imply modulation of the production of ROS by LSS75. A time course of exposure to LSS15 and LSS75 was therefore performed, with DCF added to the culture media for the last 30 min to assess ROS levels. In line with previous reports (Duerrschmidt et al., 2006), short term (4 and 8 h) exposure to LSS15 enhanced ROS production, but this response was abrogated after 24 h (Fig. 1D). By contrast exposure to LSS75 did not significantly elevate ROS production at 4 or 8 h and exposure to LSS75 for 24 h significantly lowered ROS production per cell compared to LSS15.

### Modulation of dual specificity phosphatases (DUSPs) and mitogen-activated protein kinases (MAP kinases)

**Fig. 2:**
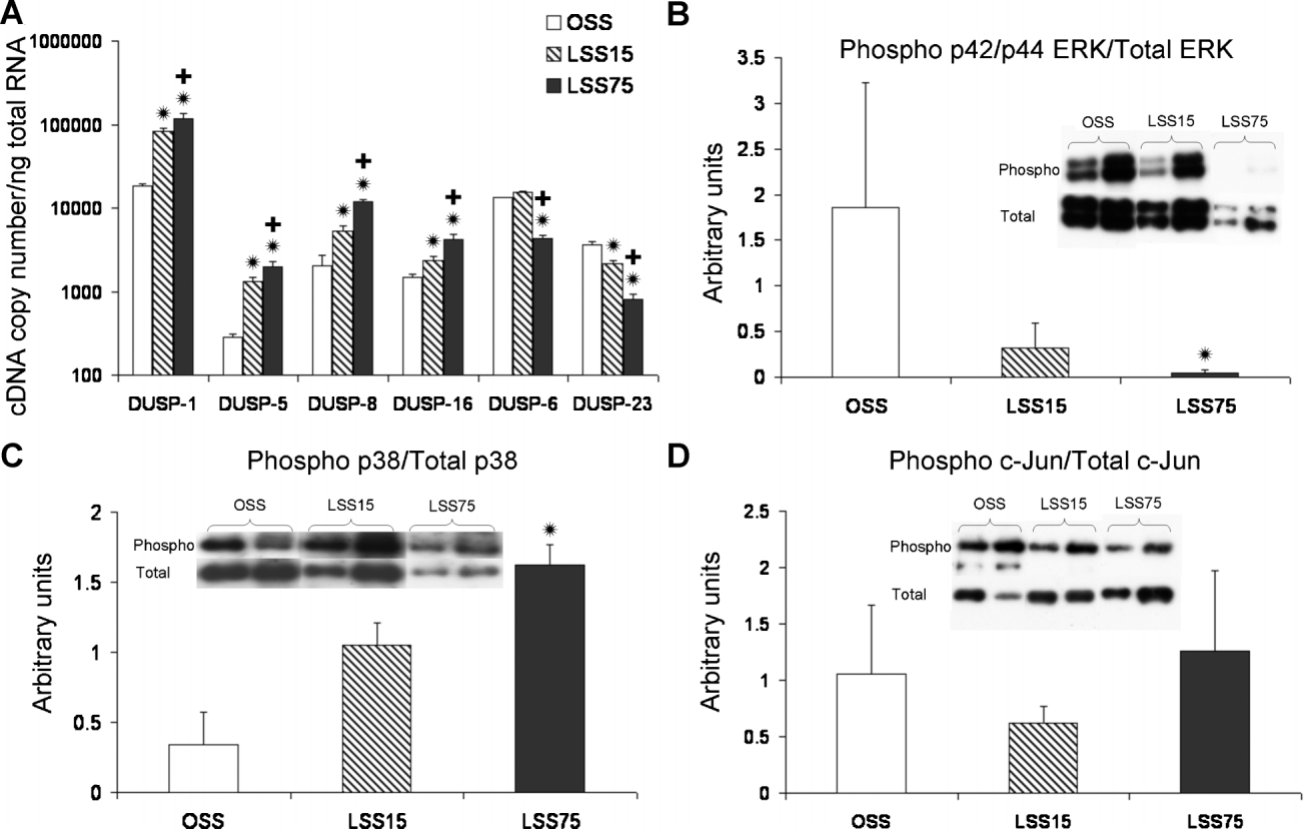
A: Changes in gene expression assayed by qPCR, 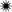 *P* < 0.05 compared to OSS; 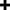 *P* < 0.05 compared to LSS15 (n = 6–9). B–D: Western blot assessment of the ratio of phosphorylated to total ERK1/2, p38 and c-Jun (n = 4, arbitrary units). 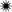 LSS75 *P* < 0.05 compared to OSS.

Members of the MAP kinase regulators, the DUSPs, were also prominent among the genes modulated by LSS75 in the array. DUSPs can dephosphorylate and therefore deactivate the MAP kinase effectors ERKs, JNK, and p38 (Patterson et al., 2009). Indeed, shear modulation of MAP kinase signaling has been documented in vitro and in vivo, via regulation of DUSP-1 (also known as MKP-1) (Zakkar et al., 2008). We found that DUSPs-1, -5, -8, and -16 mRNAs increased monophasically between OSS, LSS15, and LSS75, whereas DUSP-23 decreased; DUSP-6 decreased only between LSS15 and LSS75 (Fig. 2A). LSS75 decreased the ratio of phosphorylated p42/44 ERK (1/2) compared to OSS (Fig. 2B). By contrast however, LSS75 significantly increased the ratio of phosphorylated p38 MAP kinase (Fig. 2C). The ratio of phosphorylated c-Jun was not changed by exposure to shear for 24 h (Fig. 2D), however different batches of HUVEC tested displayed some variability in MAP kinase response including changes in total levels of expression. The levels of phosphorylated MAP kinases are subject to regulation at multiple post-transcriptional levels, including their phosphorylation and location within the cell. The level of phosphorylation is a balance of activation by upstream kinases and deactivation by DUSPs.

### Promoter analysis

Whole genome rVISTA analysis was performed to identify evolutionary conserved transcription factor binding sites modified by LSS75 (Zambon et al., 2005). This searches for sites that are conserved between the human and mouse sequence in the proximal 5 kb of promoters. The promoters of the 300 genes most upregulated and 300 most downregulated between LSS75 and LSS15 were analyzed (these genes changed more than 1.7-fold and were all significantly modulated by LSS75—*P* < 0.05).

Sixty six transcription factor binding sites were significantly enriched in the promoters of genes whose expression was significantly enhanced and four sites in the genes downregulated at LSS75 compared to LSS15. By comparing these two lists and removing those that appeared in both lists (to account for non-LSS75 regulated transcription factor binding sites) 64 sites were found only in the upregulated group and one in the downregulated group (Supplementary Table III). Nrf2 (and its related site NFE2) were in the top seven enriched sites in upregulated genes. This transcription factor binds to the anti-oxidant response element and the resulting changes in gene expression may help to explain the reduction in ROS production noted in Figure 1D above.

### Modulation of ATF and related transcription factors and cAMP levels

**Fig. 3:**
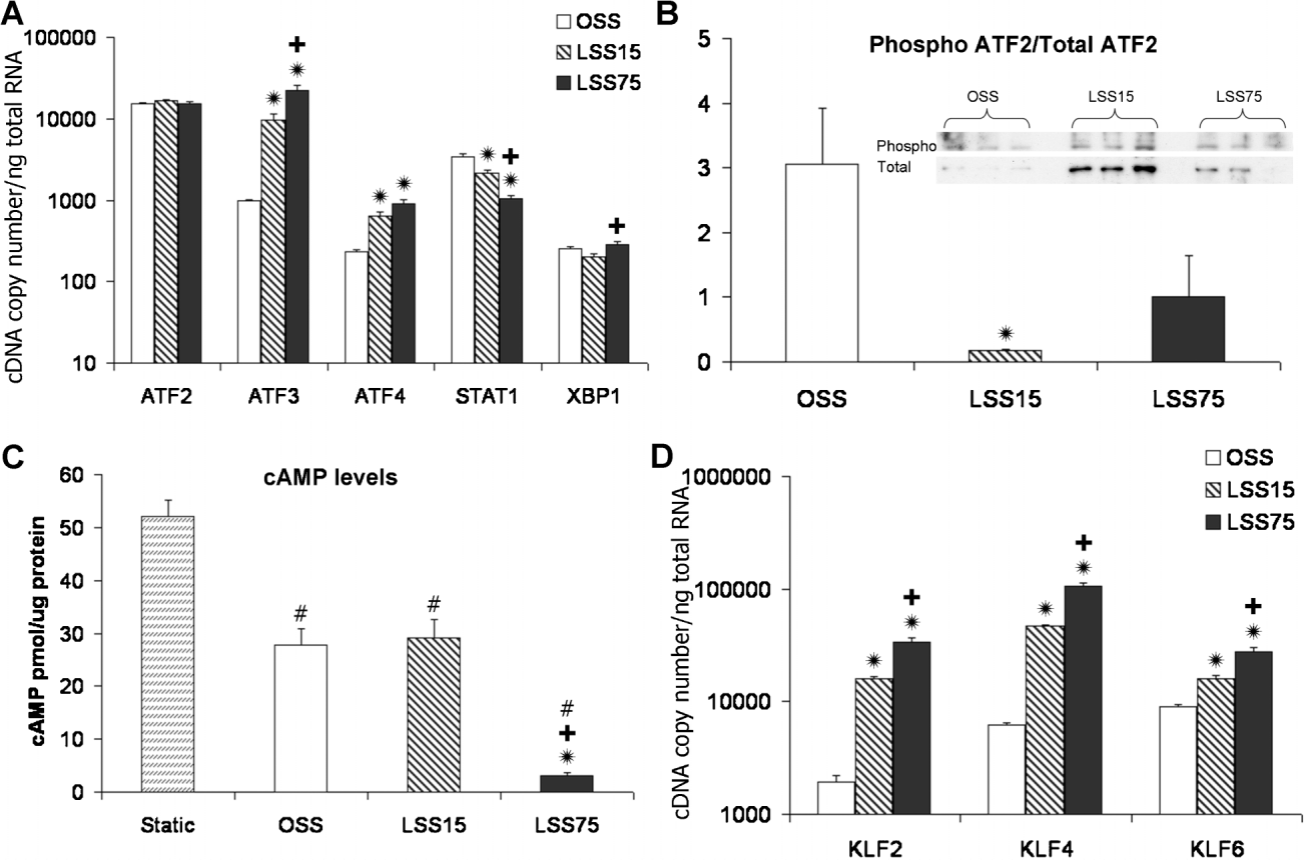
A: Changes in gene expression assayed by qPCR, 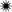 *P* < 0.05 compared to OSS; 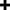 *P* < 0.05 compared to LSS15 (n = 6–9). B: Western blot assessment of the ratio of phosphorylated ATF2 to total ATF2, 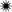 LSS15 significantly lower than OSS (n = 3, *P* < 0.01). C: cAMP levels in HUVEC ^#^*P* < 0.01 compared to static; 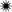 *P* < 0.01 compared to OSS; 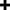 *P* < 0.01 compared to LSS15. Static cultures had significantly higher cAMP levels compared to all sheared samples, LSS75 showed significant ∼9-fold reduction in intracellular cAMP levels 16 h after commencement of shear (n = 3, *P* < 0.01). D: Changes in gene expression assayed by qPCR, 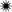 *P* < 0.05 compared to OSS; 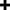 *P* < 0.05 compared to LSS15 (n = 6–9).

The rVISTA analysis identified that in the genes upregulated at LSS75, eight of the top sixteen most enriched transcription factor binding sites were for ATF transcription factors, three of which (underlined) bind ATF2 (ATF, ATF1, CREBATF, CREBP1, CREBP1CJUN, ATF3, ATF4, TAXCREB). Nevertheless, ATF2 was not found to be regulated at the transcriptional level by shear stress (Fig. 3A), however the ratio of the phosphorylated to total ATF2 was significantly reduced by exposure to LSS15 compared to OSS predominantly due to an increase in total ATF2 expression observed at LSS15 (Fig. 3B). Since several members of the ATF family of transcription factors can be activated by cyclic AMP, intracellular cAMP levels were analyzed by competitive ELISA. LSS75 induced a ninefold decrease in intracellular cAMP levels compared to OSS and LSS15 (Fig. 3C). Static cultures showed a significantly increased level of cAMP to all sheared samples.

### Other transcription factors modulated by LSS75

Also prominent among the transcription factors upregulated at LSS75 compared to LSS15 in the array were three members of the KLF family, KLF2, KLF4, and KLF6 (Fig. 3D). KLF2 has been demonstrated to be a major regulator of endothelial phenotype in response to laminar shear stress (Dekker et al., 2002; Atkins and Jain, 2007). Its expression was increased 8.2-fold between OSS and LSS15 and a further 2.1-fold between LSS15 and LSS75, which could be partly responsible for the amplification of shear responsive signaling at LSS75. KLF4 was recently demonstrated to coordinate atheroprotective gene expression programs with KLF2 (Villarreal et al., 2010). We found KLF4 to increase with magnitude similar to KLF2 (7.5-fold between OSS and LSS15, 2.3-fold LSS15 and LSS75). Consistent with this, expression of thrombomodulin, a KLF2 and KLF4 responsive gene (Dekker et al., 2006; Hamik et al., 2007), was increased 2.8-fold between OSS and LSS15 and a further 1.8-fold between LSS15 and LSS75 (*P* < 0.01). KLF6 also showed modest regulation by shear stress, increasing ∼1.7-fold between OSS, LSS15, and LSS75. In addition, STAT1 expression decreased between OSS, LSS15, and LSS75 (Fig. 3A).

### Changes in mRNA for vasoactive and inflammatory modulators

**Fig. 4:**
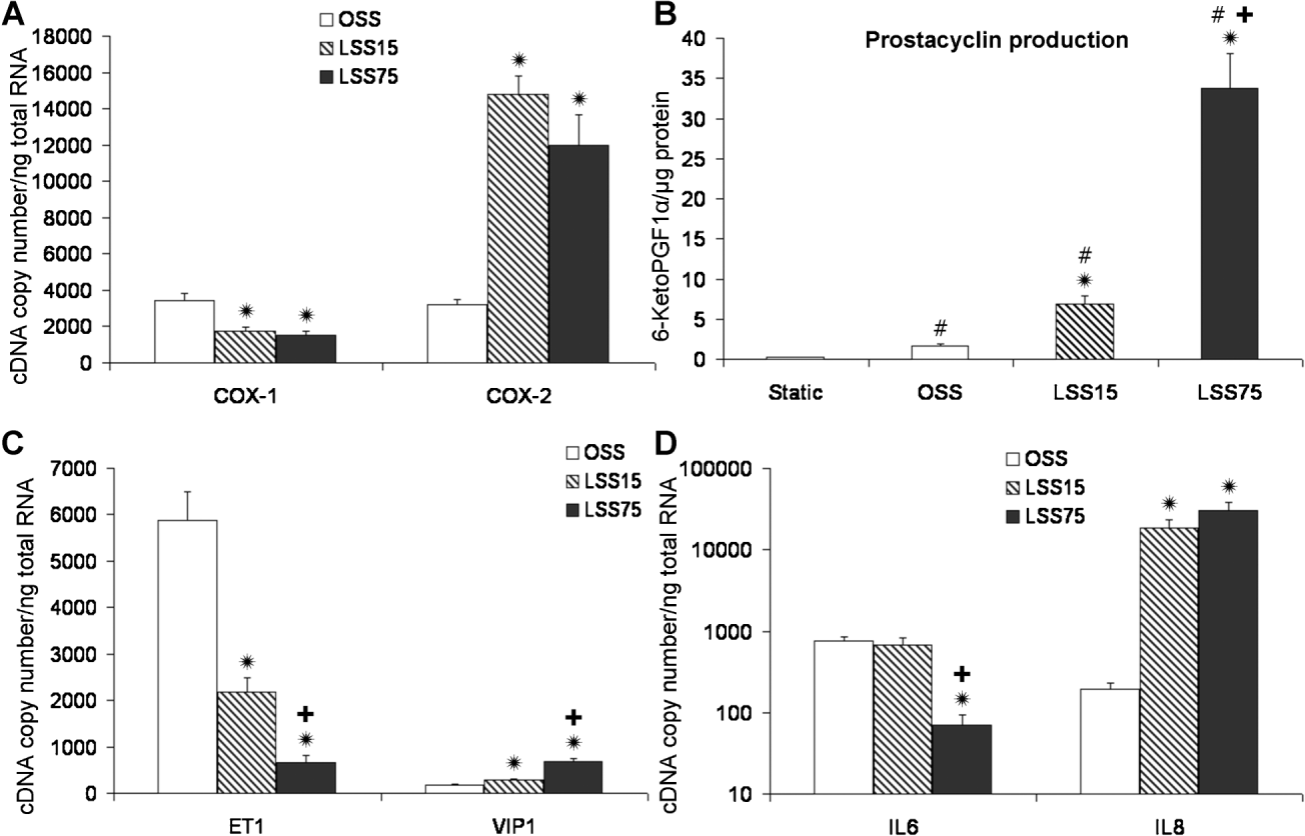
A,C,D: Changes in gene expression assayed by qPCR, 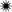 *P* < 0.05 compared to OSS; 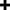 *P* < 0.05 compared to LSS15 (n = 6–9). B: Quantification of 6-keto PGF_1α_ production (the stable metabolite produced from prostacyclin-PGI_2_ breakdown) in HUVEC 16 h after commencement of shear, ^#^*P* < 0.01 compared to static; 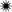 *P* < 0.01 compared to OSS; 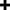 *P* < 0.01 compared to LSS15 (n = 3).

A number of changes in vasoregulatory pathways were noted in the array (Supplementary Table I). The expression of cyclooxygenases (COX1, COX2) was affected by shear stress. Under OSS, both COX1 and COX2 were expressed at similar levels (Fig. 4A) but exposure to either LSS15 or LSS75 reduced the expression of COX1, whereas it increased the level of COX2 expression approximately sevenfold. Interestingly, OSS, LSS15, and LSS75 progressively increased the production of prostacyclin compared to static cultures (Fig. 4B), part of which may be explained by the upregulation of COX2.

Changes of some vasoactive peptides were also noted. LSS15 and LSS75 caused a stepwise reduction in the mRNA expression of the vasoconstrictor peptide endothelin-1 (ET1—2.7-fold between OSS and LSS15 and 3.3-fold between LSS15 and LSS75, *P* < 0.01) and a stepwise increase in the vasodilator vasoactive intestinal peptide (VIP—1.5-fold between OSS and LSS15 and 2.4-fold between LSS15 and LSS75, *P* < 0.01; Fig. 4C).

One of the specific changes LSS75 induced in endothelial cells was a marked downregulation of interleukin-6 (IL-6) mRNA. IL-6 showed a 9.5-fold downregulation by LSS75 (*P* < 0.01) compared to OSS and LSS15 (Fig. 4D). By contrast, interleukin-8 mRNA was increased equally by exposure to LSS15 and LSS75 (Fig. 4D).

### Changes in expression of growth, migration, and angiogenesis related genes

**Fig. 5:**
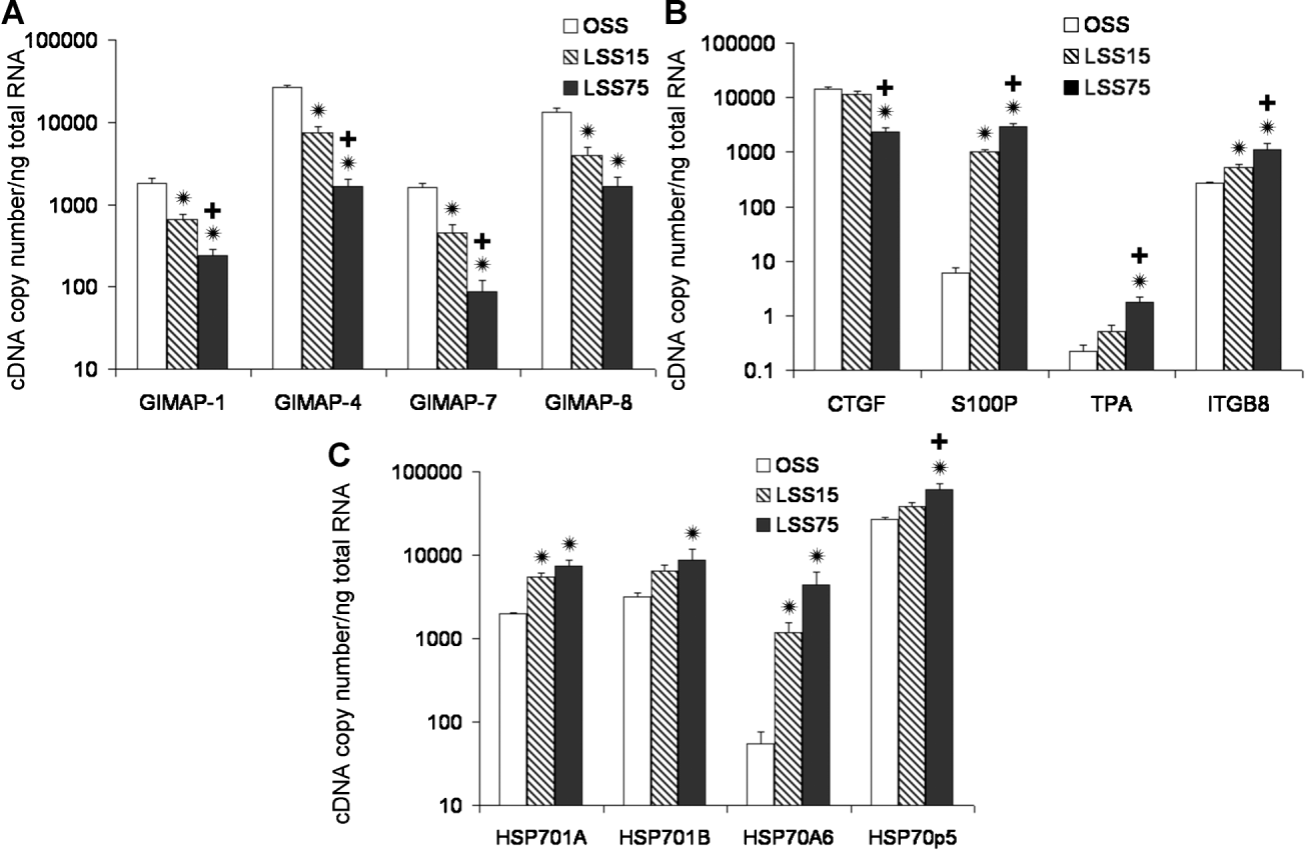
A–C: Changes in gene expression assayed by qPCR 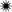 *P* < 0.05 compared to OSS; 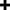 *P* < 0.05 compared to LSS15 (n = 6–9).

*G*TPase of *im*munity-*a*ssociated *p*roteins (GIMAPs) are a family of proteins know to affect T cell survival and differentiation (Nitta and Takahama, 2007), additionally they can activate NFκB via MAP kinases, however their role in endothelial cells has not been studied. GIMAP1, 4, 7, and 8 mRNA showed a monophasic reduction by LSS15 and LSS75 (Fig. 5A).

S100P is a small EF-hand Ca^2+^ binding protein that has both intracellular and extracellular action; in endothelial cells it has been shown to activate the receptor for advanced glycation end-products (RAGE) enhancing migration and proliferation (Arumugam et al., 2004; Fuentes et al., 2007). S100P mRNA expression increased 180-fold from OSS to LSS15, and a further 2.8-fold between LSS15 and LSS75. Connective tissue growth factor (CTGF) sequesters VEGF and can be cleaved by matrix metalloproteinases (MMPs), releasing VEGF to bind to its receptor (Hashimoto et al., 2002). CTGF expression was significantly reduced by LSS75 (4.9-fold, *P* < 0.01, Fig. 5B). Additionally, thrombospondin-1 which exerts an anti-angiogenic response (Zhang and Lawler, 2007) decreased 2.5-fold between OSS and LSS15 and a further 3.6-fold between LSS15 and LSS75 (*P* < 0.01; data not shown). Integrin β8 (ITGB8) binds the TGFβ latency associated peptide (LAP), facilitating MMP-14 mediated cleavage, which releases active TGFβ (Aluwihare et al., 2009). ITGB8 increased ∼2-fold between OSS and LSS15, and also between LSS15 and LSS75. Tissue plasminogen activator (TPA) expression was significantly increased at LSS75 (Fig. 5B). Finally, the heat shock protein 70 (HSP70) family of pro-survival molecular chaperones have recently been linked to the enhancement of VEGF signaling via PI3K and AKT (Hu et al., 2006; Shiota et al., 2010). Exposure to LSS potently induced the expression of HSP701A and HSP70A6, whilst LSS75 increased the mRNA expression of HSP701B and HSP70p5 (Fig. 5C). All these changes would be expected to increase the angiogenic/migratory potential of endothelial cells at LSS75.

## Discussion

This is the first study to systematically study the effect of elevated shear stress in endothelial cells and clearly demonstrates that it modifies endothelial behavior, inducing a unique transcriptomic and metabolomic response. LSS75 initiates an antioxidant phenotype in endothelial cells. LSS75 (compared to OSS and LSS15) evoked a marked upregulation of three metallothioneins (MT1X, MT1M, MT1G) by approximately tenfold at the mRNA level. MT1 and MT2A genes are highly homologous, preventing individual analysis to be performed with currently available antibodies. Further work is needed to allow their individual roles to be elucidated. LSS75 significantly upregulated the short constitutively active form of NOX5, which is of potential interest in light of the observation that NOX5 overexpression increased the activity of eNOS (Zhang et al., 2008). NOX5 may play a minor role in overall ROS production in endothelial cells as ROS levels are suppressed at LSS75 despite the observed increase in NOX5 expression. The discordant regulation of NOX5 protein compared to its mRNA level suggests it is also regulated at the level of translation or protein stability. It will be important to study the cellular location of these different molecules to understand how elevated laminar shear stress modifies ROS production and signaling, including the effect of NO.

From the gene array results, promoters were screened to identify transcription factors involved in the changes in expression observed between LSS15 and LSS75. This analysis suggested the importance of the ATF family of transcription factors as well as Nrf2; however the changes in gene expression are likely to be a combination of multiple signaling pathways (e.g. reduction in cAMP, ROS, and ERK 1/2 but an increase in p38 MAP kinase), which are modified by exposure to LSS75. The role of ATF transcription factors is of potential interest and an area for future study. As previously demonstrated, nuclear localization of ATF2 is suppressed by laminar shear (Fledderus et al., 2007; Boon et al., 2010); changes in the proportion of the phosphorylated form of ATF2 may be an additional mechanism of shear regulation. This might occur in part through the activation of p38 MAP kinase, which can activate ATF2 (Waas et al., 2001) and upregulate ATF3 (Lu et al., 2007). ATF4 can act as either a transcriptional activator or repressor depending on the presence of strong PKA activation, such that in the presence of PKA activation ATF4 represses gene transcription, but in the absence of PKA activation ATF4 activates transcription (Hai and Hartman, 2001). As cAMP levels (and intuitively PKA activation) are reduced at LSS75, it is likely to be an activator of transcription, especially as ATF4 binding sites are enriched in genes that showed increased expression at LSS75. ATF4 can also be upregulated by Nrf2 in endothelial cells (Afonyushkin et al., 2010).

The response of endothelial cells to elevated shear stress therefore appears to combine an amplification of the atheroprotective effects of laminar shear with a unique response to this stimulus, in approximately equal parts. Upregulation of metallothioneins and members of the HSP70 family, with enhanced drive through the KLF2/KLF4 axis and reduced of ERK1/2 and IL-6 signaling, could be seen as cytoprotective. These changes are balanced with reduced intracellular cAMP, increases in p38 signaling, the enhancement of ATF family signaling, which could be seen as detrimental. The effect of increased NOX5 derived ROS with its potential modification of eNOS activity is unknown in endothelial cells cultured under LSS. Further studies are needed to elucidate the function of GIMAPs in endothelial cells. The response to LSS75 may also be affected by the paracrine actions of shear-induced genes.

Chronically elevated high shear is normally resolved by expansive remodeling, however due to a failure of this response, endothelial cells overlying stenotic plaques may be exposed to very high shear for extended periods. Thus the detrimental changes induced by high shear stress may sensitize endothelial cells to additional effectors of endothelial dysfunction and combine to limit normal endothelial function with pathological results. Our experiments were restricted by technical factors to the study of shear stress experienced by moderately stenotic (∼40%) plaques. Moreover, the preparation of endothelial cells we used came from normal umbilical veins. Future studies are therefore warranted in human coronary artery endothelial cells under additional stressors, including hypercholesterolemia and increased glucose concentration. With these modifications, the conditions may more closely approximate to those associated with plaque rupture and erosion, which occur in high shear regions of stenotic plaques. For example, a recent IVUS and fluid mechanical modeling study was conducted on culprit lesions that caused a myocardial infarction or unstable angina showed that plaque rupture was associated with proximal regions of the plaques that were predicted to experience high shear stress rather than the distal, low shear stress portions of the plaques (Fukumoto et al., 2008). Endothelial erosion, that is, loss of a large patch of endothelial cells from the surface of an atherosclerotic plaque, accounts for approximately 30% of myocardial infarction (Virmani et al., 2001, 2006; Sato et al., 2005). Shear forces also play a role in plaque erosion (Lovett and Rothwell, 2003; R. Virmani, personal communication). Data presented here and future experiments may therefore shed light on these events, which are of pivotal importance for symptomatic coronary artery disease.

## References

[b1] Afonyushkin T, Oskolkova OV, Philippova M, Resink TJ, Erne P, Binder BR, Bochkov VN (2010). Oxidized phospholipids regulate expression of ATF4 and VEGF in endothelial cells via NRF2-dependent mechanism: Novel point of convergence between electrophilic and unfolded protein stress pathways. Arterioscler Thromb Vasc Biol.

[b2] Aluwihare P, Mu Z, Zhao Z, Yu D, Weinreb PH, Horan GS, Violette SM, Munger JS (2009). Mice that lack activity of {alpha}v{beta}6- and {alpha}v{beta}8-integrins reproduce the abnormalities of Tgfb1- and Tgfb3-null mice. J Cell Sci.

[b3] Arumugam T, Simeone DM, Schmidt AM, Logsdon CD (2004). S100P stimulates cell proliferation and survival via receptor for activated glycation end products (RAGE). J Biol Chem.

[b4] Atkins GB, Jain MK (2007). Role of kruppel-like transcription factors in endothelial biology. Circ Res.

[b5] Boon RA, Leyen TA, Fontijn RD, Fledderus JO, Baggen JMC, Volger OL, van Nieuw Amerongen GP, Horrevoets AJG (2010). KLF2-induced actin shear fibers control both alignment to flow and JNK signaling in vascular endothelium. Blood.

[b6] Castier Y, Ramkhelawon B, Riou S, Tedgui A, Lehoux S (2009). Role of NF-kappa B in Flow-Induced Vascular Remodeling. Antioxidants Redox Signal.

[b7] Dai GH, Kaazempur-Mofrad MR, Natarajan S, Zhang YZ, Vaughn S, Blackman BR, Kamm RD, Garcia-Cardena G, Gimbrone MA (2004). Distinct endothelial phenotypes evoked by arterial waveforms derived from atherosclerosis-susceptible and -resistant regions of human vasculature. Proc Natl Acad Sci USA.

[b8] Dai G, Vaughn S, Zhang Y, Wang ET, Garcia-Cardena G, Gimbrone MA (2007). Biomechanical forces in atherosclerosis-resistant vascular regions regulate endothelial redox balance via phosphoinositol 3-kinase/Akt-dependent activation of Nrf2. Circ Res.

[b9] Dekker RJ, van Soest S, Fontijn RD, Salamanca S, de Groot PG, VanBavel E, Pannekoek H, Horrevoets AJG (2002). Prolonged fluid shear stress induces a distinct set of endothelial cell genes, most specifically lung Kruppel-like factor (KLF2). Blood.

[b10] Dekker RJ, Boon RA, Rondaij MG, Kragt A, Volger OL, Elderkamp YW, Meijers JCM, Voorberg J, Pannekoek H, Horrevoets AJG (2006). KLF2 provokes a gene expression pattern that establishes functional quiescent differentiation of the endothelium. Blood.

[b11] Duerrschmidt N, Stielow C, Muller G, Pagano PJ, Morawietz H (2006). NO-mediated regulation of NAD(P)H oxidase by laminar shear stress in human endothelial cells. J Physiol.

[b12] Fledderus JO, van Thienen JV, Boon RA, Dekker RJ, Rohlena J, Volger OL, Bijnens A-PJJ, Daemen MJAP, Kuiper J, van Berkel TJC, Pannekoek H, Horrevoets AJG (2007). Prolonged shear stress and KLF2 suppress constitutive proinflammatory transcription through inhibition of ATF2. Blood.

[b13] Fuentes M, Nigavekar S, Arumugam T, Logsdon C, Schmidt A, Park J, Huang E (2007). RAGE activation by S100P in colon cancer stimulates growth, migration, and cell signaling pathways. Dis Colon Rectum.

[b14] Fukumoto Y, Hiro T, Fujii T, Hashimoto G, Fujimura T, Yamada J, Okamura T, Matsuzaki M (2008). Localized elevation of shear stress is related to coronary plaque rupture: A 3-dimensional intravascular ultrasound study with in-vivo color mapping of shear stress distribution. J Am Coll Cardiol.

[b15] Gentleman RC, Carey VJ, Bates DM, Bolstad B, Dettling M, Dudoit S, Ellis B, Gautier L, Ge YC, Gentry J, Hornik K, Hothorn T, Huber W, Iacus S, Irizarry R, Leisch F, Li C, Maechler M, Rossini AJ, Sawitzki G, Smith C, Smyth G, Tierney L, Yang JYH, Zhang JH (2004). Bioconductor: Open software development for computational biology and bioinformatics. Genome Biol.

[b16] Gijsen FJH, Wentzel JJ, Thury A, Mastik F, Schaar JA, Schuurbiers JCH, Slager CJ, van der Giessen WJ, de Feyter PJ, van der Steen AFW, Serruys PW (2008). Strain distribution over plaques in human coronary arteries relates to shear stress. Am J Physiol Heart Circ Physiol.

[b17] Gould KL, Lipscomb K, Hamilton GW (1974). Physiologic basis for assessing critical coronary stenosis—Instantaneous flow response and regional distribution during coronary hyperemia as measures of coronary flow reserve. Am J Cardiol.

[b18] Hai T, Hartman MG (2001). The molecular biology and nomenclature of the activating transcription factor/cAMP responsive element binding family of transcription factors: Activating transcription factor proteins and homeostasis. Gene.

[b19] Hamik A, Lin Z, Kumar A, Balcells M, Sinha S, Katz J, Feinberg MW, Gerszten RE, Edelman ER, Jain MK (2007). Kruppel-like factor 4 regulates endothelial inflammation. J Biol Chem.

[b20] Hashimoto G, Inoki I, Fujii Y, Aoki T, Ikeda E, Okada Y (2002). Matrix metalloproteinases cleave connective tissue growth factor and reactivate angiogenic activity of vascular endothelial growth factor 165. J Biol Chem.

[b21] Hu G, Tang J, Zhang B, Lin Y, Hanai J-I, Galloway J, Bedell V, Bahary N, Han Z, Ramchandran R, Thisse B, Thisse C, Zon LI, Sukhatme VP (2006). A novel endothelial-specific heat shock protein HspA12B is required in both zebrafish development and endothelial functions in vitro. J Cell Sci.

[b22] Ilegbusi O, Valaski-Tuema E, Bello F, Cotin S (2010). A fluid-structure interaction index of coronary plaque rupture. Biomedical simulation, proceedings.

[b23] Joshi AK, Leask RL, Myers JG, Ojha M, Butany J, Ethier CR (2004). Intimal thickness is not associated with wall shear stress patterns in the human right coronary artery. Arterioscler Thromb Vasc Biol.

[b24] Leach J, Rayz V, Soares B, Wintermark M, Mofrad M, Saloner D (2010). Carotid atheroma rupture observed in vivo and FSI-predicted stress distribution based on pre-rupture imaging. Ann Biomed Eng.

[b25] Lehoux S (2006). Redox signalling in vascular responses to shear and stretch. Cardiovasc Res.

[b26] Lin ZY, Kumar A, SenBanerjee S, Staniszewski K, Parmar K, Vaughan DE, Gimbrone MA, Balasubramanian V, Garcia-Cardena G, Jain MK (2005). Kruppel-like factor 2 (KLF2) regulates endothelial thrombotic function. Circ Res.

[b27] Lovett JK, Rothwell PM (2003). Site of carotid plaque ulceration in relation to direction of blood flow: An angiographic and pathological study. Cerebrovasc Dis.

[b28] Lu D, Chen J, Hai T (2007). The regulation of ATF3 gene expression by mitogen-activated protein kinases. Biochem J.

[b29] Mohan S, Mohan N, Sprague EA (1997). Differential activation of NF-kappa B in human aortic endothelial cells conditioned to specific flow environments. Am J Physiol Cell Physiol.

[b30] Mohan S, Mohan N, Valente AJ, Sprague EA (1999). Regulation of low shear flow-induced HAEC VCAM-1 expression and monocyte adhesion. Am J Physiol Cell Physiol.

[b31] Mukai Y, Wang CY, Rikitake Y, Liao JK (2007). Phosphatidylinositol 3-kinase/protein kinase Akt negatively regulates plasminogen activator inhibitor type 1 expression in vascular endothelial cells. Am J Physiol Heart Circul Physiol.

[b32] Nitta T, Takahama Y (2007). The lymphocyte guard-IANs: Regulation of lymphocyte survival by IAN/GIMAP family proteins. Trends Immunol.

[b33] Parmar KM, Larman HB, Dai GH, Zhang YH, Wang ET, Moorthy SN, Kratz JR, Lin ZY, Jain MK, Gimbrone MA, Garcia-Cardena G (2006). Integration of flow-dependent endothelial phenotypes by Kruppel-like factor 2. J Clin Invest.

[b34] Partridge J, Carlsen H, Enesa K, Chaudhury H, Zakkar M, Luong L, Kinderlerer A, Johns M, Blomhoff R, Mason JC, Haskard DO, Evans PC (2007). Laminar shear stress acts as a switch to regulate divergent functions of NF-{kappa}B in endothelial cells. FASEB J.

[b35] Patterson KI, Brummer T, O'Brien PM, Daly RJ (2009). Dual-specificity phosphatases: Critical regulators with diverse cellular targets. Biochem J.

[b36] Sato Y, Hatakeyama K, Yamashita A, Marutsuka K, Sumiyoshi A, Asada Y (2005). Proportion of fibrin and platelets differs in thrombi on ruptured and eroded coronary atherosclerotic plaques in humans. Heart.

[b37] Schirmer SH, Fledderus JO, Bot PTG, Moerland PD, Hoefer IE, Baan J, Henriques JPS, van der Schaaf RJ, Vis MM, Horrevoets AJG, Piek JJ, van Royen N (2008). Interferon-beta signaling is enhanced in patients with insufficient coronary collateral artery development and inhibits arteriogenesis in mice. Circ Res.

[b38] Shiota M, Kusakabe H, Izumi Y, Hikita Y, Nakao T, Funae Y, Miura K, Iwao H (2010). Heat shock cognate protein 70 is essential for Akt signaling in endothelial function. Arterioscler Thromb Vasc Biol.

[b39] Stone PH, Coskun AU, Kinlay S, Clark ME, Sonka M, Wahle A, Ilegbusi OJ, Yeghiazarians Y, Popma JJ, Orav J, Kuntz RE, Feldman CL (2003). Effect of endothelial shear stress on the progression of coronary artery disease, vascular remodeling, and in-stent restenosis in humans: In vivo 6-month follow-up study. Circulation.

[b40] Teng ZZ, Canton G, Yuan C, Ferguson M, Yang C, Huang XY, Zheng J, Woodard PK, Tang DL (2010). 3D Critical plaque wall stress is a better predictor of carotid plaque rupture sites than flow shear stress: An in vivo MRI-based 3D FSI study. J Biomech Eng Trans ASME.

[b41] Torii R, Wood NB, Hadjiloizou N, Dowsey AW, Wright AR, Hughes AD, Davies J, Francis DP, Mayet J, Yang GZ, Thom SAM, Xu XY (2009). Stress phase angle depicts differences in coronary artery hemodynamics due to changes in flow and geometry after percutaneous coronary intervention. Am J Physiol Heart Circul Physiol.

[b42] Villarreal G, Zhang Y, Larman HB, Gracia-Sancho J, Koo A, García-Cardeña G (2010). Defining the regulation of KLF4 expression and its downstream transcriptional targets in vascular endothelial cells. Biochem Biophys Res Commun.

[b43] Virmani R, Burke AP, Farb A (2001). Sudden cardiac death. Cardiovasc Pathol.

[b44] Virmani R, Burke AP, Farb A, Kolodgie FD (2006). Pathology of the vulnerable plaque. J Am Coll Cardiol.

[b45] Waas WF, Lo H-H, Dalby KN (2001). The kinetic mechanism of the dual phosphorylation of the ATF2 transcription factor by p38 mitogen-activated protein (MAP) kinase I. J Biol Chem.

[b46] Wentzel JJ, Janssen E, Vos J, Schuurbiers JCH, Krams R, Serruys PW, de Feyter PJ, Slager CJ (2003). Extension of increased atherosclerotic wall thickness into high shear stress regions is associated with loss of compensatory remodeling. Circulation.

[b47] Zakkar M, Chaudhury H, Sandvik G, Enesa K, Luong LA, Cuhlmann S, Mason JC, Krams R, Clark AR, Haskard DO, Evans PC (2008). Increased endothelial mitogen-activated protein kinase phosphatase-1 expression suppresses proinflammatory activation at sites that are resistant to atherosclerosis. Circ Res.

[b48] Zambon AC, Zhang L, Minovitsky S, Kanter JR, Prabhakar S, Salomonis N, Vranizan K, Dubchak I, Conklin BR, Insel PA (2005). Gene expression patterns define key transcriptional events in cell-cycle regulation by cAMP and protein kinase A. Proc Natl Acad Sci USA.

[b49] Zbinden S, Wang J, Adenika R, Schmidt M, Tilan JU, Najafi AH, Peng X, Lassance-Soares RM, Iantorno M, Morsli H, Gercenshtein L, Jang GJ, Epstein SE, Burnett MS (2010). Metallothionein enhances angiogenesis and arteriogenesis by modulating smooth muscle cell and macrophage function. Arterioscler Thromb Vasc Biol.

[b50] Zhang XF, Lawler J (2007). Thrombospondin-based antiangiogenic therapy. Microvasc Res.

[b51] Zhang Q, Malik P, Pandey D, Gupta S, Jagnandan D, de Chantemele EB, Banfi B, Marrero MB, Rudic RD, Stepp DW, Fulton DJR (2008). Paradoxical activation of endothelial nitric oxide synthase by NADPH oxidase. Arterioscler Thromb Vasc Biol.

