# Supplementary material for: Characterization of the Differential Response of Endothelial Cells Exposed to Normal and Elevated Laminar Shear Stress
Source: J Cell Physiol. 2011 Feb 1;226(11):2841–8. doi: 10.1002/jcp.22629 (PMC3412226; doi:10.1002/jcp.22629)
Supplement: Supplementary file 1 [file jcp0226-2841-SD1.doc]

Supplementary Figure I. Heat map of 50 most upregulated and 50 most downregulated genes to demonstrate the reproducibility of the results by GSEA heat map.


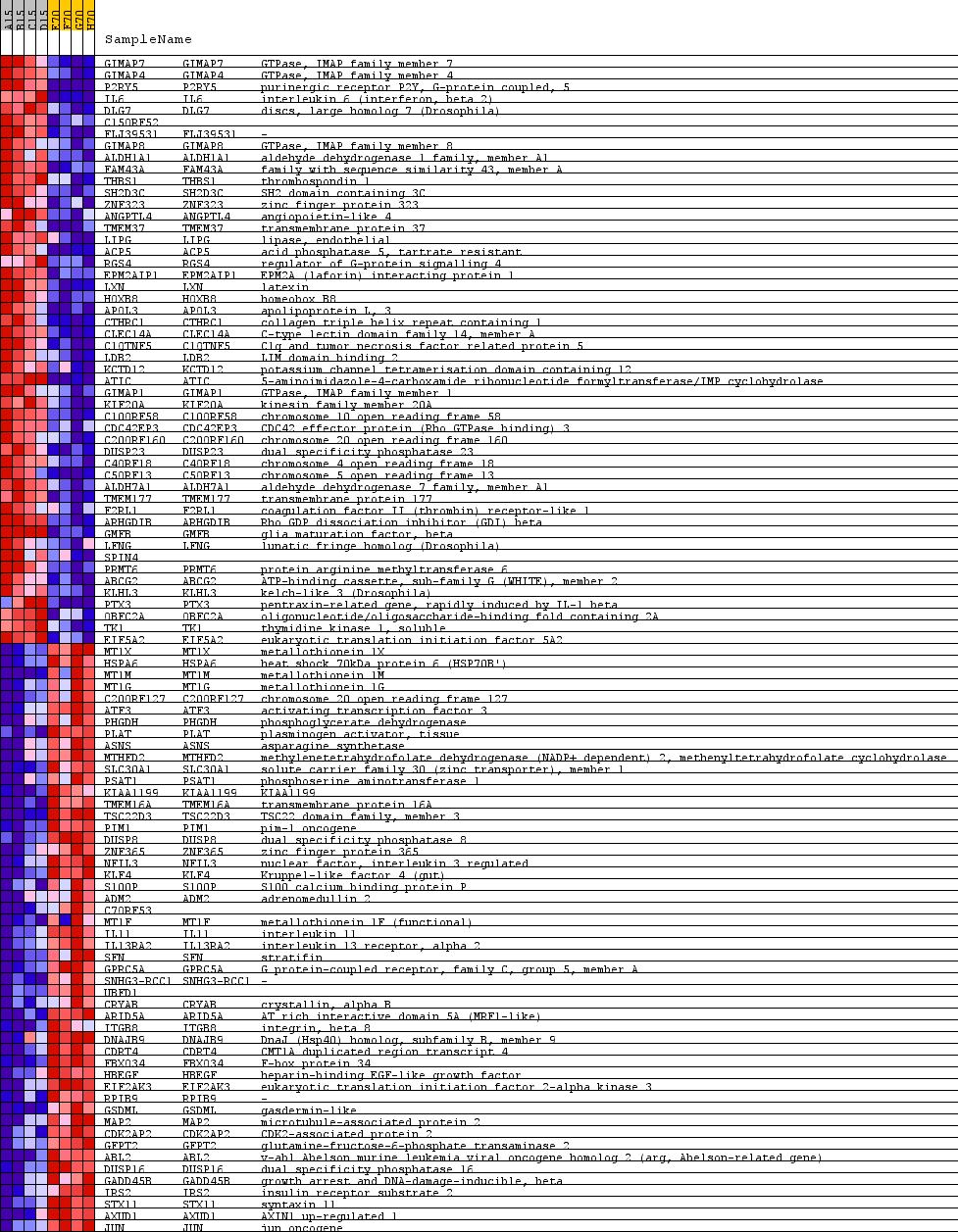


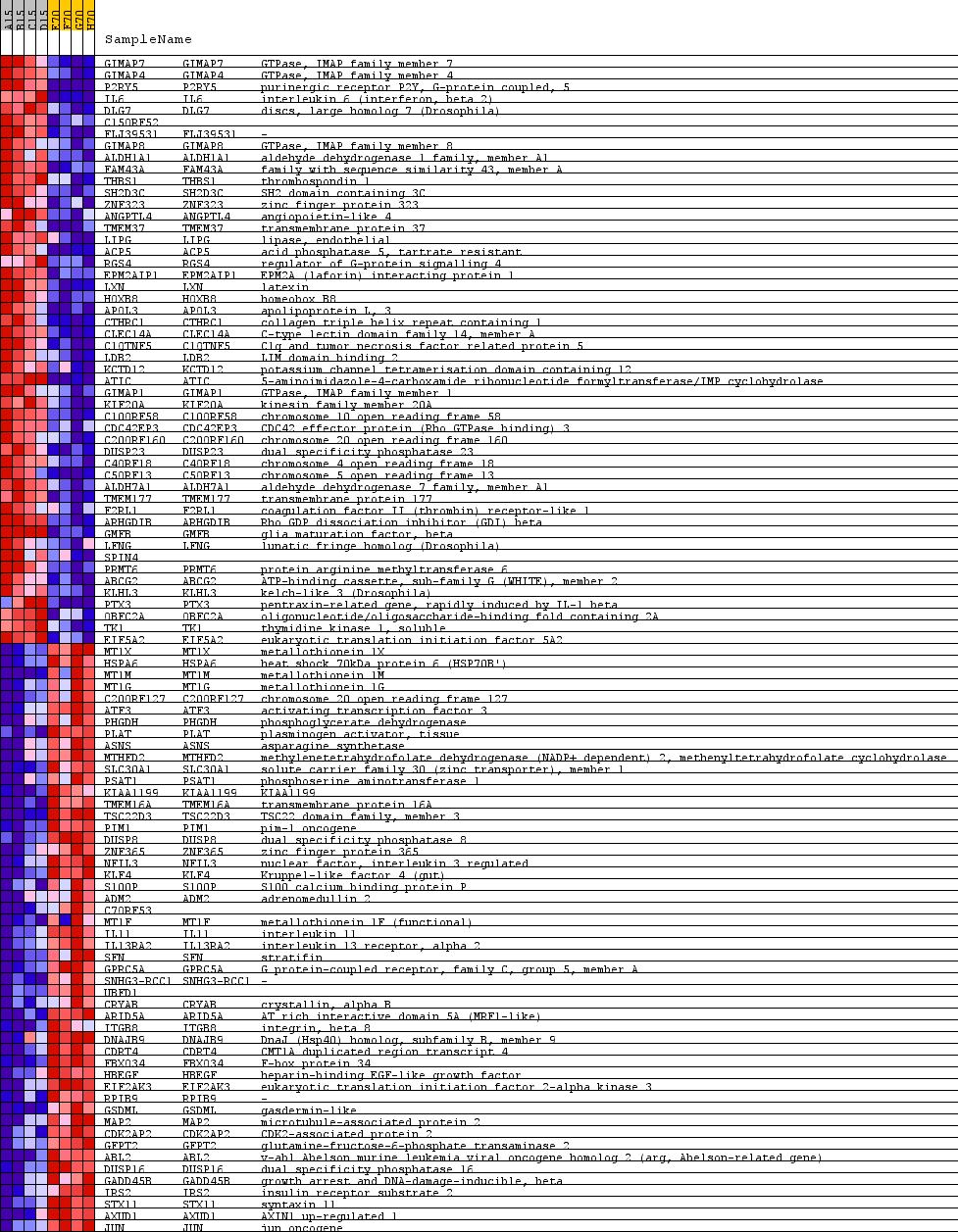


Supplementary Table I.

Results from Illumina gene array showing the genes that are upregulated or downregulated at 75 dynes/cm^2^ compared to 15 dynes/cm^2^.

Upregulated genes

| **ILMN_GENE** | **fold change** | **FDR** | **GSEA SCORE** | **DEFINITION** | **GENE_ID** |
| --- | --- | --- | --- | --- | --- |
| MT1X | 21.4 | 0.000460 | -0.98 | metallothionein 1X (MT1X), mRNA. | 4501 |
| HSPA6 | 12.6 | 0.000401 | -0.97 | heat shock 70kDa protein 6 (HSP70B') (HSPA6), mRNA. | 3310 |
| MT1G | 10.1 | 0.010564 | -0.82 | metallothionein 1G (MT1G), mRNA. | 4495 |
| MT1M | 9.4 | 0.004614 | -0.88 | metallothionein 1M (MT1M), mRNA. | 4499 |
| C20ORF127 | 7.5 | 0.005804 | -0.73 | chromosome 20 open reading frame 127 (C20orf127), mRNA. | 140851 |
| SLC30A1 | 5.4 | 0.000398 | -0.57 | solute carrier family 30 (zinc transporter), member 1 (SLC30A1), mRNA. | 7779 |
| PHGDH | 4.9 | 0.004082 | -0.62 | phosphoglycerate dehydrogenase (PHGDH), mRNA. | 26227 |
| ATF3 | 4.6 | 0.000039 | -0.65 | activating transcription factor 3 (ATF3), transcript variant 3, mRNA. | 467 |
| MTHFD2 | 4.5 | 0.000241 | -0.58 | methylenetetrahydrofolate dehydrogenase (NADP+ dependent) 2, methenyltetrahydrofolate cyclohydrolase (MTHFD2). | 10797 |
| ASNS | 4.3 | 0.001036 | -0.58 | asparagine synthetase (ASNS), transcript variant 1, mRNA. | 440 |
| PLAT | 4.2 | 0.000017 | -0.60 | plasminogen activator, tissue (PLAT), transcript variant 1, mRNA. | 5327 |
| TSC22D3 | 4.2 | 0.000017 | -0.50 | TSC22 domain family, member 3 (TSC22D3), transcript variant 3, mRNA. | 1831 |
| PIM1 | 4.1 | 0.000060 | -0.50 | pim-1 oncogene (PIM1), mRNA. | 5292 |
| NFIL3 | 3.8 | 0.000013 | -0.47 | nuclear factor, interleukin 3 regulated (NFIL3), mRNA. | 4783 |
| PSAT1 | 3.8 | 0.013418 | -0.57 | phosphoserine aminotransferase 1 (PSAT1), transcript variant 2, mRNA. | 29968 |
| KIAA1199 | 3.8 | 0.001015 | -0.56 | KIAA1199 (KIAA1199), mRNA. | 57214 |
| KLF4 | 3.6 | 0.000016 | -0.45 | Kruppel-like factor 4 (gut) (KLF4), mRNA. | 9314 |
| S100P | 3.5 | 0.002639 | -0.45 | S100 calcium binding protein P (S100P), mRNA. | 6286 |
| MT1F | 3.2 | 0.059068 | -0.43 | metallothionein 1F (MT1F), mRNA. | 4494 |
| HSPA1B | 3.2 | 0.000287 | -0.35 | heat shock 70kDa protein 1B (HSPA1B), mRNA. | 3304 |
| DUSP8 | 3.2 | 0.000016 | -0.49 | dual specificity phosphatase 8 (DUSP8), mRNA. | 1850 |
| SNHG3-RCC1 | 3.1 | 0.004086 | -0.40 | regulator of chromosome condensation 1 (SNHG3-RCC1), transcript variant 1, mRNA. | 751867 |
| FBXO34 | 3.1 | 0.000014 | -0.38 | F-box protein 34 (FBXO34), mRNA. | 55030 |
| TMEM16A | 3.1 | 0.000204 | -0.54 | transmembrane protein 16A (TMEM16A), mRNA. | 55107 |
| ZNF364 | 3.0 | 0.000052 | -0.34 | zinc finger protein 364 (ZNF364), mRNA. | 27246 |
| STX11 | 2.9 | 0.000015 | -0.37 | syntaxin 11 (STX11), mRNA. | 8676 |
| AXUD1 | 2.9 | 0.000037 | -0.36 | AXIN1 up-regulated 1 (AXUD1), mRNA. | 64651 |
| ZNF365 | 2.9 | 0.003120 | -0.48 | zinc finger protein 365 (ZNF365), transcript variant A, mRNA. | 22891 |
| ADM2 | 2.8 | 0.027308 | -0.44 | adrenomedullin 2 (ADM2), mRNA. | 79924 |
| STX1A | 2.8 | 0.000016 | -0.36 | syntaxin 1A (brain) (STX1A), mRNA. | 6804 |
| UBFD1 | 2.8 | 0.000400 | -0.40 | ubiquitin family domain containing 1 (UBFD1), mRNA. | 56061 |
| DNAJB9 | 2.7 | 0.000180 | -0.38 | DnaJ (Hsp40) homolog, subfamily B, member 9 (DNAJB9), mRNA. | 4189 |
| PHLDA1 | 2.7 | 0.000036 | -0.32 | pleckstrin homology-like domain, family A, member 1 (PHLDA1), mRNA. | 22822 |
| C7ORF53 | 2.7 | 0.016197 | -0.43 | chromosome 7 open reading frame 53 (C7orf53), mRNA. | 286006 |
| IL13RA2 | 2.7 | 0.001103 | -0.41 | interleukin 13 receptor, alpha 2 (IL13RA2), mRNA. | 3598 |
| DDIT4 | 2.7 | 0.027433 | -0.28 | DNA-damage-inducible transcript 4 (DDIT4), mRNA. | 54541 |
| HSPA1A | 2.7 | 0.004085 | -0.27 | heat shock 70kDa protein 1A (HSPA1A), mRNA. | 3303 |
| HEY1 | 2.7 | 0.016184 | -0.33 | hairy/enhancer-of-split related with YRPW motif 1 (HEY1), transcript variant 2, mRNA. | 23462 |
| XBP1 | 2.6 | 0.000149 | -0.35 | X-box binding protein 1 (XBP1), transcript variant 1, mRNA. | 7494 |
| CDR2 | 2.6 | 0.000036 | -0.31 | cerebellar degeneration-related protein 2, 62kDa (CDR2), mRNA. | 1039 |
| BAMBI | 2.6 | 0.000760 | -0.34 | BMP and activin membrane-bound inhibitor homolog (Xenopus laevis) (BAMBI), mRNA. | 25805 |
| GADD45A | 2.6 | 0.000137 | -0.32 | growth arrest and DNA-damage-inducible, alpha (GADD45A), mRNA. | 1647 |
| CRYAB | 2.6 | 0.025169 | -0.39 | crystallin, alpha B (CRYAB), mRNA. | 1410 |
| CDK2AP2 | 2.6 | 0.000147 | -0.37 | CDK2-associated protein 2 (CDK2AP2), mRNA. | 10263 |
| THBD | 2.6 | 0.000184 | -0.35 | thrombomodulin (THBD), mRNA. | 7056 |
| DUSP5 | 2.5 | 0.000402 | -0.28 | dual specificity phosphatase 5 (DUSP5), mRNA. | 1847 |
| C13ORF15 | 2.5 | 0.000104 | -0.27 | chromosome 13 open reading frame 15 (C13orf15), mRNA. | 28984 |
| MAP2 | 2.5 | 0.000480 | -0.37 | microtubule-associated protein 2 (MAP2), transcript variant 2, mRNA. | 4133 |
| VIP | 2.5 | 0.001713 | -0.35 | vasoactive intestinal peptide (VIP), transcript variant 2, mRNA. | 7432 |
| HBEGF | 2.5 | 0.000431 | -0.38 | heparin-binding EGF-like growth factor (HBEGF), mRNA. | 1839 |
| PLA2G4C | 2.5 | 0.031931 | -0.31 | phospholipase A2, group IVC (cytosolic, calcium-independent) (PLA2G4C), mRNA. | 8605 |
| IL11 | 2.5 | 0.000071 | -0.41 | interleukin 11 (IL11), mRNA. | 3589 |
| CDRT4 | 2.5 | 0.000145 | -0.38 | CMT1A duplicated region transcript 4 (CDRT4), mRNA. | 284040 |
| GSDML | 2.4 | 0.003689 | -0.37 | gasdermin-like (GSDML), transcript variant 1, mRNA. | 55876 |
| IBRDC3 | 2.4 | 0.000122 | -0.34 | IBR domain containing 3 (IBRDC3), mRNA. | 127544 |
| ELL2 | 2.4 | 0.000149 | -0.35 | elongation factor, RNA polymerase II, 2 (ELL2), mRNA. | 22936 |
| IRS2 | 2.4 | 0.000477 | -0.37 | insulin receptor substrate 2 (IRS2), mRNA. | 8660 |
| AYTL2 | 2.4 | 0.000059 | -0.35 | acyltransferase like 2 (AYTL2), mRNA. | 79888 |
| GFPT2 | 2.4 | 0.000387 | -0.37 | glutamine-fructose-6-phosphate transaminase 2 (GFPT2), mRNA. | 9945 |
| HYOU1 | 2.4 | 0.000941 | -0.33 | hypoxia up-regulated 1 (HYOU1), mRNA. | 10525 |
| CNKSR3 | 2.4 | 0.000307 | -0.32 | CNKSR family member 3 (CNKSR3), mRNA. | 154043 |
| GADD45B | 2.4 | 0.001948 | -0.37 | growth arrest and DNA-damage-inducible, beta (GADD45B), mRNA. | 4616 |
| ANXA3 | 2.4 | 0.028553 | -0.34 | annexin A3 (ANXA3), mRNA. | 306 |
| ABL2 | 2.4 | 0.000148 | -0.37 | v-abl Abelson murine leukemia viral oncogene homolog 2 (arg, Abelson-related gene) (ABL2), transcript variant b, mRNA. | 27 |
| SFN | 2.4 | 0.001182 | -0.41 | stratifin (SFN), mRNA. | 2810 |
| EIF2AK3 | 2.4 | 0.000063 | -0.38 | eukaryotic translation initiation factor 2-alpha kinase 3 (EIF2AK3), mRNA. | 9451 |
| ITGB8 | 2.4 | 0.004891 | -0.38 | integrin, beta 8 (ITGB8), mRNA. | 3696 |
| CRTAC1 | 2.3 | 0.000215 | -0.36 | cartilage acidic protein 1 (CRTAC1), mRNA. | 55118 |
| LMCD1 | 2.3 | 0.000098 | -0.34 | LIM and cysteine-rich domains 1 (LMCD1), mRNA. | 29995 |
| SIAH2 | 2.3 | 0.001535 | -0.33 | seven in absentia homolog 2 (Drosophila) (SIAH2), mRNA. | 6478 |
| RND3 | 2.3 | 0.002428 | -0.29 | Rho family GTPase 3 (RND3), mRNA. | 390 |
| GPRC5A | 2.3 | 0.000094 | -0.41 | G protein-coupled receptor, family C, group 5, member A (GPRC5A), mRNA. | 9052 |
| KCNK1 | 2.3 | 0.001723 | -0.36 | potassium channel, subfamily K, member 1 (KCNK1), mRNA. | 3775 |
| NIPA1 | 2.3 | 0.000183 | -0.31 | non imprinted in Prader-Willi/Angelman syndrome 1 (NIPA1), mRNA. | 123606 |
| DUSP1 | 2.3 | 0.001358 | -0.26 | dual specificity phosphatase 1 (DUSP1), mRNA. | 1843 |
| MYCT1 | 2.3 | 0.000034 | -0.26 | myc target 1 (MYCT1), mRNA. | 80177 |
| C12ORF48 | 2.3 | 0.005314 | -0.25 | chromosome 12 open reading frame 48 (C12orf48), mRNA. | 55010 |
| TMEM170 | 2.3 | 0.000169 | -0.31 | transmembrane protein 170 (TMEM170), mRNA. | 124491 |
| IL8 | 2.3 | 0.000286 | -0.30 | interleukin 8 (IL8), mRNA. | 3576 |
| NDRG1 | 2.3 | 0.000065 | -0.23 | N-myc downstream regulated gene 1 (NDRG1), mRNA. | 10397 |
| TICAM2 | 2.3 | 0.001214 | -0.32 | toll-like receptor adaptor molecule 2 (TICAM2), mRNA. | 353376 |
| HSPA5 | 2.3 | 0.001134 | -0.30 | heat shock 70kDa protein 5 (glucose-regulated protein, 78kDa) (HSPA5), mRNA. | 3309 |
| JUN | 2.3 | 0.000061 | -0.36 | jun oncogene (JUN), mRNA. | 3725 |
| C1ORF63 | 2.2 | 0.000503 | -0.28 | chromosome 1 open reading frame 63 (C1orf63), transcript variant 1, mRNA. | 57035 |
| DNAJB1 | 2.2 | 0.008349 | -0.25 | DnaJ (Hsp40) homolog, subfamily B, member 1 (DNAJB1), mRNA. | 3337 |
| NR2C2 | 2.2 | 0.000413 | -0.32 | nuclear receptor subfamily 2, group C, member 2 (NR2C2), mRNA. | 7182 |
| ARID5A | 2.2 | 0.000061 | -0.39 | AT rich interactive domain 5A (MRF1-like) (ARID5A), mRNA. | 10865 |
| KLF6 | 2.2 | 0.000089 | -0.22 | Kruppel-like factor 6 (KLF6), transcript variant 2, mRNA. | 1316 |
| DUSP16 | 2.2 | 0.000061 | -0.37 | dual specificity phosphatase 16 (DUSP16), mRNA. | 80824 |
| KLHL21 | 2.2 | 0.000277 | -0.26 | kelch-like 21 (Drosophila) (KLHL21), mRNA. | 9903 |
| SLC39A14 | 2.2 | 0.000227 | -0.32 | solute carrier family 39 (zinc transporter), member 14 (SLC39A14), mRNA. | 23516 |
| RORA | 2.2 | 0.000175 | -0.33 | RAR-related orphan receptor A (RORA), transcript variant 2, mRNA. | 6095 |
| OGT | 2.2 | 0.000092 | -0.29 | O-linked N-acetylglucosamine (GlcNAc) transferase (UDP-N-acetylglucosamine:polypeptide-N-acetylglucosaminyl transferase) (OGT) | 8473 |
| ALPL | 2.2 | 0.048905 | -0.28 | alkaline phosphatase, liver/bone/kidney (ALPL), mRNA. | 249 |
| PIP5K2A | 2.2 | 0.000102 | -0.31 | phosphatidylinositol-4-phosphate 5-kinase, type II, alpha (PIP5K2A), mRNA. | 5305 |
| PHF13 | 2.2 | 0.000123 | -0.25 | PHD finger protein 13 (PHF13), mRNA. | 148479 |
| JMY | 2.2 | 0.000211 | -0.34 | junction-mediating and regulatory protein (JMY), mRNA. | 133746 |
| RSC1A1 | 2.1 | 0.002849 | -0.30 | regulatory solute carrier protein, family 1, member 1 (RSC1A1), mRNA. | 6248 |
| RBM33 | 2.1 | 0.000240 | -0.32 | RNA binding motif protein 33 (RBM33), transcript variant 1, mRNA. | 155435 |
| CLK1 | 2.1 | 0.000876 | -0.25 | CDC-like kinase 1 (CLK1), mRNA. | 1195 |
| RPIB9 | 2.1 | 0.000243 | -0.38 | Rap2-binding protein 9 (RPIB9), mRNA. | 154661 |
| CBS | 2.1 | 0.050079 | -0.26 | cystathionine-beta-synthase (CBS), mRNA. | 875 |
| EIF4A2 | 2.1 | 0.000210 | -0.21 | eukaryotic translation initiation factor 4A, isoform 2 (EIF4A2), mRNA. | 1974 |
| MT1E | 2.1 | 0.003168 | -0.35 | metallothionein 1E (functional) (MT1E), mRNA. | 4493 |
| PDIA4 | 2.1 | 0.000241 | -0.25 | protein disulfide isomerase family A, member 4 (PDIA4), mRNA. | 9601 |
| HMGB2 | 2.1 | 0.001038 | -0.27 | high-mobility group box 2 (HMGB2), mRNA. | 3148 |
| SFRS17A | 2.1 | 0.000150 | -0.30 | splicing factor, arginine/serine-rich 17A (SFRS17A), mRNA. | 8227 |
| DKK1 | 2.1 | 0.026756 | -0.34 | dickkopf homolog 1 (Xenopus laevis) (DKK1), mRNA. | 22943 |
| TMEM158 | 2.1 | 0.000982 | -0.23 | transmembrane protein 158 (TMEM158), mRNA. | 25907 |
| ANKRD15 | 2.1 | 0.000313 | -0.29 | ankyrin repeat domain 15 (ANKRD15), transcript variant 2, mRNA. | 23189 |
| ZFP36 | 2.1 | 0.001037 | -0.25 | zinc finger protein 36, C3H type, homolog (mouse) (ZFP36), mRNA. | 7538 |
| RNASE4 | 2.1 | 0.000147 | -0.28 | ribonuclease, RNase A family, 4 (RNASE4), transcript variant 3, mRNA. | 6038 |
| NANS | 2.1 | 0.000394 | -0.25 | N-acetylneuraminic acid synthase (sialic acid synthase) (NANS), mRNA. | 54187 |
| CASZ1 | 2.1 | 0.000436 | -0.34 | castor zinc finger 1 (CASZ1), transcript variant 2, mRNA. | 54897 |
| HK2 | 2.1 | 0.002148 | -0.33 | hexokinase 2 (HK2), mRNA. | 3099 |
| POFUT2 | 2.1 | 0.000147 | -0.27 | protein O-fucosyltransferase 2 (POFUT2), transcript variant 1, mRNA. | 23275 |
| GEM | 2.1 | 0.004364 | -0.33 | GTP binding protein overexpressed in skeletal muscle (GEM), transcript variant 1, mRNA. | 2669 |
| XPC | 2.1 | 0.000416 | -0.25 | xeroderma pigmentosum, complementation group C (XPC), mRNA. | 7508 |
| CDYL2 | 2.0 | 0.000271 | -0.30 | chromodomain protein, Y-like 2 (CDYL2), mRNA. | 124359 |
| LOC196752 | 2.0 | 0.000290 | -0.32 | similar to CG32542-PA (LOC196752), mRNA. | 196752 |
| DMRTA1 | 2.0 | 0.007880 | -0.28 | DMRT-like family A1 (DMRTA1), mRNA. | 63951 |
| HIST1H2AC | 2.0 | 0.019714 | -0.30 | histone cluster 1, H2ac (HIST1H2AC), mRNA. | 8334 |
| RSRC2 | 2.0 | 0.000152 | -0.23 | arginine/serine-rich coiled-coil 2 (RSRC2), transcript variant 3, mRNA. | 65117 |
| ZBTB2 | 2.0 | 0.000401 | -0.30 | zinc finger and BTB domain containing 2 (ZBTB2), mRNA. | 57621 |
| ST3GAL1 | 2.0 | 0.001677 | -0.24 | ST3 beta-galactoside alpha-2,3-sialyltransferase 1 (ST3GAL1), transcript variant 1, mRNA. | 6482 |
| SENP5 | 2.0 | 0.000223 | -0.28 | SUMO1/sentrin specific peptidase 5 (SENP5), mRNA. | 205564 |
| EPB41L4A | 2.0 | 0.000450 | -0.31 | erythrocyte membrane protein band 4.1 like 4A (EPB41L4A), mRNA. | 64097 |
| RYBP | 2.0 | 0.001109 | -0.26 | RING1 and YY1 binding protein (RYBP), mRNA. | 23429 |
| ARL5B | 2.0 | 0.000653 | -0.30 | ADP-ribosylation factor-like 5B (ARL5B), mRNA. | 221079 |
| FAM84B | 2.0 | 0.000970 | -0.28 | family with sequence similarity 84, member B (FAM84B), mRNA. | 157638 |
| C6ORF145 | 2.0 | 0.000604 | -0.28 | PREDICTED: chromosome 6 open reading frame 145 (C6orf145), mRNA. | 221749 |
| SERPINB2 | 2.0 | 0.009219 | -0.20 | serpin peptidase inhibitor, clade B (ovalbumin), member 2 (SERPINB2), mRNA. | 5055 |
| DNAJB2 | 2.0 | 0.000694 | -0.23 | DnaJ (Hsp40) homolog, subfamily B, member 2 (DNAJB2), transcript variant 2, mRNA. | 3300 |
| FEM1B | 2.0 | 0.000312 | -0.31 | fem-1 homolog b (C. elegans) (FEM1B), mRNA. | 10116 |
| ARIH1 | 2.0 | 0.000256 | -0.24 | ariadne homolog, ubiquitin-conjugating enzyme E2 binding protein, 1 (Drosophila) (ARIH1), mRNA. | 25820 |
| KLHL15 | 2.0 | 0.000199 | -0.33 | kelch-like 15 (Drosophila) (KLHL15), mRNA. | 80311 |
| TICAM1 | 2.0 | 0.000201 | -0.24 | toll-like receptor adaptor molecule 1 (TICAM1), transcript variant 2, mRNA. | 148022 |
| NUCB2 | 2.0 | 0.000995 | -0.22 | nucleobindin 2 (NUCB2), mRNA. | 4925 |
| ERRFI1 | 2.0 | 0.002764 | -0.22 | ERBB receptor feedback inhibitor 1 (ERRFI1), mRNA. | 54206 |
| CSNK1E | 2.0 | 0.000215 | -0.26 | casein kinase 1, epsilon (CSNK1E), transcript variant 2, mRNA. | 1454 |
| CLCF1 | 2.0 | 0.000468 | -0.27 | cardiotrophin-like cytokine factor 1 (CLCF1), mRNA. | 23529 |
| LOC153222 | 2.0 | 0.000359 | -0.28 | adult retina protein (LOC153222), mRNA. | 153222 |
| LOC731786 | 2.0 | 0.001903 | -0.22 | PREDICTED: similar to 60S ribosomal protein L32 (LOC731786), mRNA. | 731786 |
| SHMT2 | 2.0 | 0.005683 | -0.21 | serine hydroxymethyltransferase 2 (mitochondrial) (SHMT2), mRNA. | 6472 |
| NENF | 2.0 | 0.036552 | -0.26 | neuron derived neurotrophic factor (NENF), mRNA. | 29937 |

Downregulated genes

| **ILMN_GENE** | **fold change** | **FDR** | **GSEA SCORE** | **DEFINITION** | **GENE_ID** |
| --- | --- | --- | --- | --- | --- |
| GIMAP4 | -10.6 | 0.000291 | 0.82 | GTPase, IMAP family member 4 (GIMAP4), mRNA. | 55303 |
| GIMAP7 | -8.9 | 0.000810 | 0.85 | GTPase, IMAP family member 7 (GIMAP7), mRNA. | 168537 |
| THBS1 | -4.6 | 0.000058 | 0.49 | thrombospondin 1 (THBS1), mRNA. | 7057 |
| P2RY5 | -4.5 | 0.000199 | 0.70 | purinergic receptor P2Y, G-protein coupled, 5 (P2RY5), mRNA. | 10161 |
| GIMAP8 | -4.3 | 0.002643 | 0.52 | GTPase, IMAP family member 8 (GIMAP8), mRNA. | 155038 |
| FAM43A | -4.1 | 0.000059 | 0.49 | family with sequence similarity 43, member A (FAM43A), mRNA. | 131583 |
| ALDH1A1 | -4.0 | 0.000416 | 0.50 | aldehyde dehydrogenase 1 family, member A1 (ALDH1A1), mRNA. | 216 |
| LDB2 | -3.8 | 0.005063 | 0.41 | LIM domain binding 2 (LDB2), mRNA. | 9079 |
| C10ORF58 | -3.7 | 0.000017 | 0.40 | chromosome 10 open reading frame 58 (C10orf58), mRNA. | 84293 |
| C15ORF52 | -3.6 | 0.000444 | 0.53 | chromosome 15 open reading frame 52 (C15orf52), mRNA. | 388115 |
| ARHGDIB | -3.6 | 0.000011 | 0.37 | Rho GDP dissociation inhibitor (GDI) beta (ARHGDIB), mRNA. | 397 |
| IL6 | -3.5 | 0.001105 | 0.58 | interleukin 6 (interferon, beta 2) (IL6), mRNA. | 3569 |
| RGS4 | -3.4 | 0.017812 | 0.44 | regulator of G-protein signalling 4 (RGS4), mRNA. | 5999 |
| LIPG | -3.3 | 0.000470 | 0.46 | lipase, endothelial (LIPG), mRNA. | 9388 |
| ANGPTL4 | -3.3 | 0.000176 | 0.47 | angiopoietin-like 4 (ANGPTL4), transcript variant 1, mRNA. | 51129 |
| KCTD12 | -3.3 | 0.001096 | 0.41 | potassium channel tetramerisation domain containing 12 (KCTD12), mRNA. | 115207 |
| MFNG | -3.2 | 0.000058 | 0.35 | MFNG O-fucosylpeptide 3-beta-N-acetylglucosaminyltransferase (MFNG), mRNA. | 4242 |
| FLJ39531 | -3.2 | 0.000046 | 0.52 | FLJ39531 protein (FLJ39531), mRNA. | 400360 |
| SH2D3C | -3.2 | 0.000222 | 0.49 | SH2 domain containing 3C (SH2D3C), transcript variant 1, mRNA. | 10044 |
| C1QTNF5 | -3.2 | 0.000337 | 0.41 | C1q and tumor necrosis factor related protein 5 (C1QTNF5), mRNA. | 114902 |
| DLG7 | -3.2 | 0.000061 | 0.54 | discs, large homolog 7 (Drosophila) (DLG7), mRNA. | 9787 |
| ZNF323 | -3.1 | 0.001723 | 0.49 | zinc finger protein 323 (ZNF323), transcript variant 1, mRNA. | 64288 |
| LXN | -3.1 | 0.000358 | 0.43 | latexin (LXN), mRNA. | 56925 |
| C4ORF18 | -2.9 | 0.000496 | 0.39 | chromosome 4 open reading frame 18 (C4orf18), transcript variant 2, mRNA. | 51313 |
| CLEC14A | -2.9 | 0.000234 | 0.42 | C-type lectin domain family 14, member A (CLEC14A), mRNA. | 161198 |
| CTHRC1 | -2.8 | 0.001749 | 0.42 | collagen triple helix repeat containing 1 (CTHRC1), mRNA. | 115908 |
| DUSP23 | -2.8 | 0.000164 | 0.39 | dual specificity phosphatase 23 (DUSP23), mRNA. | 54935 |
| NOX4 | -2.7 | 0.000340 | 0.35 | NADPH oxidase 4 (NOX4), mRNA. | 50507 |
| GIMAP1 | -2.7 | 0.001725 | 0.41 | GTPase, IMAP family member 1 (GIMAP1), mRNA. | 170575 |
| TMEM37 | -2.7 | 0.001204 | 0.47 | transmembrane protein 37 (TMEM37), mRNA. | 140738 |
| CTGF | -2.7 | 0.000220 | 0.27 | connective tissue growth factor (CTGF), mRNA. | 1490 |
| C20ORF160 | -2.7 | 0.002535 | 0.40 | chromosome 20 open reading frame 160 (C20orf160), mRNA. | 140706 |
| GPX1 | -2.6 | 0.000119 | 0.31 | glutathione peroxidase 1 (GPX1), transcript variant 2, mRNA. | 2876 |
| ATIC | -2.6 | 0.000021 | 0.41 | 5-aminoimidazole-4-carboxamide ribonucleotide formyltransferase/IMP cyclohydrolase (ATIC), mRNA. | 471 |
| EPM2AIP1 | -2.6 | 0.000047 | 0.44 | EPM2A (laforin) interacting protein 1 (EPM2AIP1), mRNA. | 9852 |
| C5ORF13 | -2.5 | 0.002503 | 0.39 | chromosome 5 open reading frame 13 (C5orf13), mRNA. | 9315 |
| PTX3 | -2.5 | 0.002557 | 0.36 | pentraxin-related gene, rapidly induced by IL-1 beta (PTX3), mRNA. | 5806 |
| CDC42EP3 | -2.5 | 0.000174 | 0.40 | CDC42 effector protein (Rho GTPase binding) 3 (CDC42EP3), mRNA. | 10602 |
| APOL3 | -2.5 | 0.003834 | 0.42 | apolipoprotein L, 3 (APOL3), transcript variant beta/a, mRNA. | 80833 |
| HOXB8 | -2.5 | 0.000678 | 0.42 | homeobox B8 (HOXB8), mRNA. | 3218 |
| ACP5 | -2.5 | 0.001859 | 0.44 | acid phosphatase 5, tartrate resistant (ACP5), mRNA. | 54 |
| CYP1A1 | -2.5 | 0.052965 | 0.28 | cytochrome P450, family 1, subfamily A, polypeptide 1 (CYP1A1), mRNA. | 1543 |
| IGFBP4 | -2.5 | 0.013124 | 0.26 | insulin-like growth factor binding protein 4 (IGFBP4), mRNA. | 3487 |
| ASF1A | -2.5 | 0.000046 | 0.34 | ASF1 anti-silencing function 1 homolog A (S. cerevisiae) (ASF1A), mRNA. | 25842 |
| PURB | -2.4 | 0.000153 | 0.34 | purine-rich element binding protein B (PURB), mRNA. | 5814 |
| RIN2 | -2.4 | 0.001108 | 0.33 | Ras and Rab interactor 2 (RIN2), mRNA. | 54453 |
| TEAD2 | -2.4 | 0.000248 | 0.29 | TEA domain family member 2 (TEAD2), mRNA. | 8463 |
| OBFC2A | -2.4 | 0.000352 | 0.36 | oligonucleotide/oligosaccharide-binding fold containing 2A (OBFC2A), mRNA. | 64859 |
| TFPI2 | -2.4 | 0.003473 | 0.30 | tissue factor pathway inhibitor 2 (TFPI2), mRNA. | 7980 |
| GMFB | -2.4 | 0.000035 | 0.37 | glia maturation factor, beta (GMFB), mRNA. | 2764 |
| HOXA5 | -2.4 | 0.001420 | 0.29 | homeobox A5 (HOXA5), mRNA. | 3202 |
| LFNG | -2.4 | 0.011114 | 0.37 | LFNG O-fucosylpeptide 3-beta-N-acetylglucosaminyltransferase (LFNG), transcript variant 1, mRNA. | 3955 |
| F2RL1 | -2.4 | 0.003112 | 0.38 | coagulation factor II (thrombin) receptor-like 1 (F2RL1), mRNA. | 2150 |
| FABP5 | -2.3 | 0.036463 | 0.28 | fatty acid binding protein 5 (psoriasis-associated) (FABP5), mRNA. | 2171 |
| ALDH7A1 | -2.3 | 0.000152 | 0.38 | aldehyde dehydrogenase 7 family, member A1 (ALDH7A1), mRNA. | 501 |
| SPIN4 | -2.3 | 0.001776 | 0.37 | spindlin family, member 4 (SPIN4), mRNA. | 139886 |
| SLC27A3 | -2.3 | 0.000166 | 0.33 | solute carrier family 27 (fatty acid transporter), member 3 (SLC27A3), mRNA. | 11000 |
| NME1 | -2.3 | 0.000242 | 0.28 | non-metastatic cells 1, protein (NM23A) expressed in (NME1), transcript variant 1, mRNA. | 4830 |
| C16ORF33 | -2.3 | 0.000292 | 0.34 | chromosome 16 open reading frame 33 (C16orf33), mRNA. | 79622 |
| RFX5 | -2.3 | 0.000060 | 0.35 | regulatory factor X, 5 (influences HLA class II expression) (RFX5), transcript variant 2, mRNA. | 5993 |
| TMEM46 | -2.3 | 0.001359 | 0.35 | transmembrane protein 46 (TMEM46), mRNA. | 387914 |
| ETFB | -2.3 | 0.000153 | 0.34 | electron-transfer-flavoprotein, beta polypeptide (ETFB), transcript variant 2, mRNA. | 2109 |
| BCL6B | -2.3 | 0.002924 | 0.30 | B-cell CLL/lymphoma 6, member B (zinc finger protein) (BCL6B), mRNA. | 255877 |
| PRMT6 | -2.3 | 0.000336 | 0.37 | protein arginine methyltransferase 6 (PRMT6), mRNA. | 55170 |
| TUBA1A | -2.3 | 0.000075 | 0.23 | tubulin, alpha 1a (TUBA1A), mRNA. | 7846 |
| KIF20A | -2.2 | 0.000062 | 0.41 | kinesin family member 20A (KIF20A), mRNA. | 10112 |
| DIXDC1 | -2.2 | 0.001031 | 0.34 | DIX domain containing 1 (DIXDC1), transcript variant 1, mRNA. | 85458 |
| PUS7 | -2.2 | 0.000676 | 0.34 | pseudouridylate synthase 7 homolog (S. cerevisiae) (PUS7), mRNA. | 54517 |
| TRIM22 | -2.2 | 0.001560 | 0.32 | tripartite motif-containing 22 (TRIM22), mRNA. | 10346 |
| TGM2 | -2.2 | 0.000705 | 0.35 | transglutaminase 2 (C polypeptide, protein-glutamine-gamma-glutamyltransferase) (TGM2), transcript variant 2, mRNA. | 7052 |
| LOC728069 | -2.2 | 0.000835 | 0.33 | PREDICTED: similar to T-box 1 isoform C (LOC728069), mRNA. | 728069 |
| RPUSD2 | -2.2 | 0.000096 | 0.34 | RNA pseudouridylate synthase domain containing 2 (RPUSD2), mRNA. | 27079 |
| MAFB | -2.2 | 0.001141 | 0.34 | v-maf musculoaponeurotic fibrosarcoma oncogene homolog B (avian) (MAFB), mRNA. | 9935 |
| DSCR2 | -2.2 | 0.000217 | 0.29 | Down syndrome critical region gene 2 (DSCR2), transcript variant 2, mRNA. | 8624 |
| TK1 | -2.2 | 0.000515 | 0.36 | thymidine kinase 1, soluble (TK1), mRNA. | 7083 |
| VGLL4 | -2.2 | 0.000367 | 0.27 | vestigial like 4 (Drosophila) (VGLL4), mRNA. | 9686 |
| LYSMD2 | -2.2 | 0.001846 | 0.33 | LysM, putative peptidoglycan-binding, domain containing 2 (LYSMD2), mRNA. | 256586 |
| F2RL2 | -2.2 | 0.000106 | 0.35 | coagulation factor II (thrombin) receptor-like 2 (F2RL2), mRNA. | 2151 |
| GOLSYN | -2.2 | 0.002167 | 0.35 | Golgi-localized protein (GOLSYN), transcript variant 7, mRNA. | 55638 |
| ACAA2 | -2.2 | 0.000124 | 0.31 | acetyl-Coenzyme A acyltransferase 2 (mitochondrial 3-oxoacyl-Coenzyme A thiolase) | 10449 |
| RPP25 | -2.2 | 0.003076 | 0.29 | ribonuclease P/MRP 25kDa subunit (RPP25), mRNA. | 54913 |
| LYN | -2.2 | 0.009587 | 0.24 | v-yes-1 Yamaguchi sarcoma viral related oncogene homolog (LYN), mRNA. | 4067 |
| TMEM177 | -2.2 | 0.000153 | 0.38 | transmembrane protein 177 (TMEM177), mRNA. | 80775 |
| EGFL7 | -2.2 | 0.000704 | 0.28 | EGF-like-domain, multiple 7 (EGFL7), transcript variant 2, mRNA. | 51162 |
| LPXN | -2.2 | 0.000527 | 0.28 | leupaxin (LPXN), mRNA. | 9404 |
| MAT2A | -2.2 | 0.000289 | 0.26 | methionine adenosyltransferase II, alpha (MAT2A), mRNA. | 4144 |
| DOLK | -2.1 | 0.000291 | 0.31 | dolichol kinase (DOLK), mRNA. | 22845 |
| ADAMTS4 | -2.1 | 0.001489 | 0.31 | ADAM metallopeptidase with thrombospondin type 1 motif, 4 (ADAMTS4), mRNA. | 9507 |
| RANBP6 | -2.1 | 0.000239 | 0.31 | RAN binding protein 6 (RANBP6), mRNA. | 26953 |
| NOV | -2.1 | 0.003937 | 0.28 | nephroblastoma overexpressed gene (NOV), mRNA. | 4856 |
| EIF4H | -2.1 | 0.000557 | 0.31 | eukaryotic translation initiation factor 4H (EIF4H), transcript variant 2, mRNA. | 7458 |
| TNFAIP8L1 | -2.1 | 0.002780 | 0.34 | tumor necrosis factor, alpha-induced protein 8-like 1 (TNFAIP8L1), mRNA. | 126282 |
| EDN1 | -2.1 | 0.006366 | 0.27 | endothelin 1 (EDN1), mRNA. | 1906 |
| CYYR1 | -2.1 | 0.002191 | 0.33 | cysteine/tyrosine-rich 1 (CYYR1), mRNA. | 116159 |
| ATP6V0E2 | -2.1 | 0.000389 | 0.31 | ATPase, H+ transporting V0 subunit e2 (ATP6V0E2), transcript variant 1, mRNA. | 155066 |
| KLHL3 | -2.1 | 0.000497 | 0.36 | kelch-like 3 (Drosophila) (KLHL3), mRNA. | 26249 |
| PAFAH1B1 | -2.1 | 0.003422 | 0.27 | platelet-activating factor acetylhydrolase, isoform Ib, alpha subunit 45kDa (PAFAH1B1), mRNA. | 5048 |
| FAM36A | -2.1 | 0.000064 | 0.33 | family with sequence similarity 36, member A (FAM36A), mRNA. | 116228 |
| PIK3R1 | -2.1 | 0.000298 | 0.34 | phosphoinositide-3-kinase, regulatory subunit 1 (p85 alpha) (PIK3R1), transcript variant 1, mRNA. | 5295 |
| TRAPPC2L | -2.1 | 0.000436 | 0.24 | trafficking protein particle complex 2-like (TRAPPC2L), mRNA. | 51693 |
| PIGW | -2.1 | 0.000339 | 0.34 | phosphatidylinositol glycan anchor biosynthesis, class W (PIGW), mRNA. | 284098 |
| RHOBTB3 | -2.1 | 0.000959 | 0.31 | Rho-related BTB domain containing 3 (RHOBTB3), mRNA. | 22836 |
| ISG15 | -2.1 | 0.000973 | 0.28 | ISG15 ubiquitin-like modifier (ISG15), mRNA. | 9636 |
| LCMT2 | -2.1 | 0.000152 | 0.34 | leucine carboxyl methyltransferase 2 (LCMT2), mRNA. | 9836 |
| FZD4 | -2.1 | 0.001326 | 0.25 | frizzled homolog 4 (Drosophila) (FZD4), mRNA. | 8322 |
| C3ORF54 | -2.1 | 0.008159 | 0.28 | chromosome 3 open reading frame 54 (C3orf54), mRNA. | 389119 |
| FAM107A | -2.1 | 0.002452 | 0.34 | family with sequence similarity 107, member A (FAM107A), transcript variant 2, mRNA. | 11170 |
| DUSP6 | -2.1 | 0.001371 | 0.32 | dual specificity phosphatase 6 (DUSP6), transcript variant 1, mRNA. | 1848 |
| C20ORF108 | -2.1 | 0.001331 | 0.27 | chromosome 20 open reading frame 108 (C20orf108), mRNA. | 116151 |
| DBNL | -2.1 | 0.000149 | 0.25 | drebrin-like (DBNL), transcript variant 2, mRNA. | 28988 |
| NMRAL1 | -2.1 | 0.000214 | 0.27 | NmrA-like family domain containing 1 (NMRAL1), mRNA. | 57407 |
| RNASE1 | -2.1 | 0.005026 | 0.20 | ribonuclease, RNase A family, 1 (pancreatic) (RNASE1), transcript variant 2, mRNA. | 6035 |
| FRMD4A | -2.1 | 0.005438 | 0.28 | FERM domain containing 4A (FRMD4A), mRNA. | 55691 |
| CGNL1 | -2.1 | 0.018427 | 0.25 | cingulin-like 1 (CGNL1), mRNA. | 84952 |
| TSPAN6 | -2.1 | 0.001130 | 0.28 | tetraspanin 6 (TSPAN6), mRNA. | 7105 |
| KDR | -2.0 | 0.005723 | 0.31 | kinase insert domain receptor (a type III receptor tyrosine kinase) (KDR), mRNA. | 3791 |
| SMARCE1 | -2.0 | 0.000205 | 0.29 | SWI/SNF related, matrix associated, actin dependent regulator of chromatin, subfamily e, member 1 (SMARCE1), mRNA. | 6605 |
| PRKCDBP | -2.0 | 0.000106 | 0.22 | protein kinase C, delta binding protein (PRKCDBP), mRNA. | 112464 |
| TOMM34 | -2.0 | 0.000681 | 0.27 | translocase of outer mitochondrial membrane 34 (TOMM34), nuclear gene encoding mitochondrial protein, mRNA. | 10953 |
| CBX2 | -2.0 | 0.005059 | 0.32 | chromobox homolog 2 (Pc class homolog, Drosophila) (CBX2), transcript variant 2, mRNA. | 84733 |
| PSMB10 | -2.0 | 0.001050 | 0.25 | proteasome (prosome, macropain) subunit, beta type, 10 (PSMB10), mRNA. | 5699 |
| GALK1 | -2.0 | 0.000169 | 0.27 | galactokinase 1 (GALK1), mRNA. | 2584 |
| DERA | -2.0 | 0.000339 | 0.24 | 2-deoxyribose-5-phosphate aldolase homolog (C. elegans) (DERA), mRNA. | 51071 |
| ZNF792 | -2.0 | 0.001667 | 0.32 | zinc finger protein 792 (ZNF792), mRNA. | 126375 |
| ALDH3A1 | -2.0 | 0.028685 | 0.27 | aldehyde dehydrogenase 3 family, memberA1 (ALDH3A1), mRNA. | 218 |
| ABCG2 | -2.0 | 0.001313 | 0.36 | ATP-binding cassette, sub-family G (WHITE), member 2 (ABCG2), mRNA. | 9429 |
| TSEN2 | -2.0 | 0.000048 | 0.35 | tRNA splicing endonuclease 2 homolog (S. cerevisiae) (TSEN2), mRNA. | 80746 |
| PEX11B | -2.0 | 0.000462 | 0.26 | peroxisomal biogenesis factor 11B (PEX11B), mRNA. | 8799 |
| GRPEL2 | -2.0 | 0.001370 | 0.25 | GrpE-like 2, mitochondrial (E. coli) (GRPEL2), nuclear gene encoding mitochondrial protein, mRNA. | 134266 |
| MYH10 | -2.0 | 0.000691 | 0.28 | myosin, heavy chain 10, non-muscle (MYH10), mRNA. | 4628 |
| C6ORF192 | -2.0 | 0.000503 | 0.32 | chromosome 6 open reading frame 192 (C6orf192), mRNA. | 116843 |
| MYEOV | -2.0 | 0.000061 | 0.35 | myeloma overexpressed gene (in a subset of t(11;14) positive multiple myelomas) (MYEOV), mRNA. | 26579 |
| TMEM14B | -2.0 | 0.000400 | 0.24 | transmembrane protein 14B (TMEM14B), mRNA. | 81853 |
| TUT1 | -2.0 | 0.000733 | 0.29 | terminal uridylyl transferase 1, U6 snRNA-specific (TUT1), mRNA. | 64852 |
| NDRG4 | -2.0 | 0.003220 | 0.28 | NDRG family member 4 (NDRG4), mRNA. | 65009 |
| HECW2 | -2.0 | 0.006871 | 0.23 | HECT, C2 and WW domain containing E3 ubiquitin protein ligase 2 (HECW2), mRNA. | 57520 |
| MEF2C | -2.0 | 0.001534 | 0.34 | myocyte enhancer factor 2C (MEF2C), mRNA. | 4208 |
| TPM2 | -2.0 | 0.001748 | 0.22 | tropomyosin 2 (beta) (TPM2), transcript variant 2, mRNA. | 7169 |
| C1ORF212 | -2.0 | 0.000259 | 0.29 | chromosome 1 open reading frame 212 (C1orf212), mRNA. | 113444 |
| TSPAN8 | -2.0 | 0.088480 | 0.26 | tetraspanin 8 (TSPAN8), mRNA. | 7103 |
| TP53INP1 | -2.0 | 0.002579 | 0.25 | tumor protein p53 inducible nuclear protein 1 (TP53INP1), mRNA. | 94241 |
| PPP1R16B | -2.0 | 0.003959 | 0.32 | protein phosphatase 1, regulatory (inhibitor) subunit 16B (PPP1R16B), mRNA. | 26051 |
| CEP55 | -2.0 | 0.002579 | 0.30 | centrosomal protein 55kDa (CEP55), mRNA. | 55165 |
| APEX1 | -2.0 | 0.000389 | 0.23 | APEX nuclease (multifunctional DNA repair enzyme) 1 (APEX1), transcript variant 3, mRNA. | 328 |
| ST3GAL5 | -2.0 | 0.006726 | 0.31 | ST3 beta-galactoside alpha-2,3-sialyltransferase 5 (ST3GAL5), transcript variant 2, mRNA. | 8869 |
| UBLCP1 | -2.0 | 0.000151 | 0.25 | ubiquitin-like domain containing CTD phosphatase 1 (UBLCP1), mRNA. | 134510 |
| RENBP | -2.0 | 0.001631 | 0.30 | renin binding protein (RENBP), mRNA. | 5973 |
| C13ORF23 | -2.0 | 0.000874 | 0.29 | chromosome 13 open reading frame 23 (C13orf23), transcript variant 1, mRNA. | 80209 |
| EIF5A2 | -2.0 | 0.000037 | 0.36 | eukaryotic translation initiation factor 5A2 (EIF5A2), mRNA. | 56648 |
| C17ORF58 | -2.0 | 0.000398 | 0.32 | chromosome 17 open reading frame 58 (C17orf58), transcript variant 2, mRNA. | 284018 |
| MUM1 | -2.0 | 0.001364 | 0.27 | melanoma associated antigen (mutated) 1 (MUM1), mRNA. | 84939 |
| NUDT14 | -2.0 | 0.001529 | 0.21 | nudix (nucleoside diphosphate linked moiety X)-type motif 14 (NUDT14), mRNA. | 256281 |
| DPYSL3 | -2.0 | 0.001234 | 0.21 | dihydropyrimidinase-like 3 (DPYSL3), mRNA. | 1809 |
| STMN1 | -2.0 | 0.001730 | 0.30 | stathmin 1/oncoprotein 18 (STMN1), transcript variant 1, mRNA. | 3925 |

Supplementary Figure II. Quantification of mRNA and GAPDH protein levels indicated that GAPDH expression was affected by shear stress, therefore it was not used to normalise Western blots (🟊 p<0.05 compared to LSS15; # p<0.05 compared to OSS).


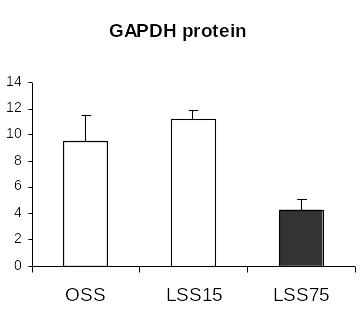


*

#

*

Copy number/ng RNA

Arbitrary units


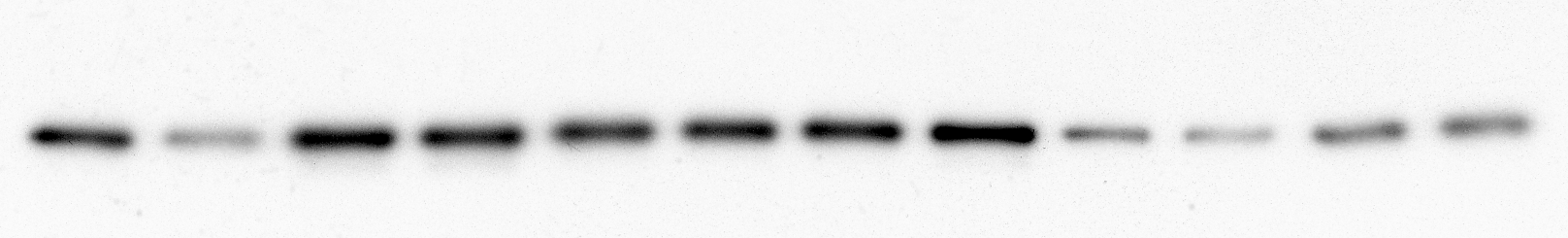


OSS

LSS15

LSS75

GAPDH

Supplementary table II. Gene expression change patterns. Monophasic (significant stepwise changes between OSS and LSS15 and LSS75). LSS75 specific changes indicate that there was no significant difference between OSS and LSS15, however there was a significant change between LSS75 and both OSS and LSS15. OSS different indicates there was a significant change between OSS and both LSS15 and LSS75, but no significant difference between LSS15 and LSS75.

| Monophasic increase | Monophasic decrease | LSS75 specifically increases expression | LSS75 specifically decreases expression | OSS different from LSS15 and LSS75 | Other |
| --- | --- | --- | --- | --- | --- |
| DUSP1 | DUSP23 | MT1G | DUSP6 | COX1 | XBP1 |
| DUSP5 | STAT1 | MT1X | IL6 | COX2 | NOX2 |
| DUSP8 | NOX4 | MT1M | CTGF | NOX5 | HSP701B |
| DUSP16 | MT1E | TPA | SNRK | p47phox | HO |
| KLF2 | ET1 | HSP70p5 |  | MT1A | NOS3 |
| KLF4 | GIMAP1 |  |  | MT1F | SLC30A1 |
| KLF6 | GIMAP4 |  |  | MT2A | ATF2 |
| ATF3 | GIMAP7 |  |  | IL8 |  |
| ATF4 | GIMAP8 |  |  | HSP701A |  |
| ZNF364 | TSP1 |  |  | HSP70A6 |  |
| p67phox | GPX1 |  |  | TPA |  |
| VIP |  |  |  | UPAR |  |
| S100P |  |  |  | CTSL1 |  |
| ITGB8 |  |  |  | SMAD7 |  |
| THMB |  |  |  |  |  |
| ASNS |  |  |  |  |  |

Supplementary table III. a) Transcription factor binding sites significantly enriched in the promoters of the 300 most upregulated genes

| **name** | **number of hits**  **in the submitted regions** | **total number of**  **hits on genome** | **log_10_(p-value)** |
| --- | --- | --- | --- |
| ATF | 101 | 3416 | 18.554 |
| ATF4 | 436 | 26282 | 18.466 |
| NRF2 | 500 | 32616 | 15.043 |
| E2F1DP1 | 565 | 38173 | 14.196 |
| MINI20 | 570 | 38710 | 13.935 |
| CREBATF | 299 | 17756 | 13.728 |
| GLI | 635 | 44154 | 13.555 |
| NFE2 | 206 | 11215 | 12.98 |
| MYC | 367 | 23703 | 11.828 |
| ATF1 | 129 | 6207 | 11.775 |
| ATF3 | 262 | 15751 | 11.618 |
| E2F1DP2 | 497 | 34433 | 11.045 |
| CREBP1CJUN | 76 | 3183 | 9.8072 |
| NFKAPPAB | 356 | 23956 | 9.4947 |
| CREBP1 | 61 | 2363 | 9.2771 |
| TAXCREB | 42 | 1325 | 9.1034 |
| ERR1 | 581 | 42698 | 8.9149 |
| RBPJK | 655 | 49039 | 8.7271 |
| HIF1 | 151 | 8663 | 8.3009 |
| SP3 | 408 | 28919 | 8.1375 |
| VBP | 256 | 16840 | 7.8715 |
| HES1 | 367 | 25756 | 7.8356 |
| DR3 | 546 | 40723 | 7.6291 |
| IK1 | 331 | 22989 | 7.5687 |
| HNF4ALPHA | 611 | 46399 | 7.393 |
| PITX2 | 636 | 48560 | 7.3481 |
| NMYC | 264 | 17846 | 7.1189 |
| AP4 | 554 | 41839 | 7.0686 |
| ZNF219 | 155 | 9536 | 6.6626 |
| RFX1 | 631 | 48905 | 6.4801 |
| FOXP3 | 553 | 42427 | 6.2689 |
| CIZ | 158 | 9945 | 6.2096 |
| POU1F1 | 527 | 40305 | 6.1618 |
| FOXO1 | 563 | 43570 | 5.9458 |
| XBP1 | 78 | 4130 | 5.885 |
| RORA1 | 73 | 3791 | 5.848 |
| ZTA | 120 | 7222 | 5.7784 |
| E4F1 | 22 | 647 | 5.6082 |
| SMAD3 | 390 | 29403 | 5.2934 |
| SOX5 | 564 | 44277 | 5.2901 |
| T3R | 23 | 730 | 5.2888 |
| SOX9 | 362 | 27130 | 5.1795 |
| IK3 | 136 | 8744 | 5.0225 |
| TFIIA | 529 | 41523 | 5.0198 |
| ARP1 | 240 | 17149 | 4.9175 |
| CEBP | 260 | 18937 | 4.7087 |
| E2 | 118 | 7487 | 4.697 |
| GATA3 | 511 | 40302 | 4.6833 |
| CHX10 | 148 | 9846 | 4.6719 |
| S8 | 230 | 16494 | 4.6514 |
| TFIII | 323 | 24258 | 4.6432 |
| STAF | 358 | 27302 | 4.5435 |
| HFH1 | 135 | 8925 | 4.4478 |
| MEIS1 | 358 | 27385 | 4.4449 |
| HP1SITEFACTOR | 242 | 17707 | 4.3184 |
| TCF4 | 174 | 12126 | 4.2985 |
| E47 | 206 | 14763 | 4.2634 |
| BACH1 | 14 | 376 | 4.2226 |
| FOXO4 | 433 | 34073 | 4.1676 |
| IRF1 | 373 | 28928 | 4.1468 |
| AREB6 | 322 | 24574 | 4.1449 |
| EGR1 | 60 | 3338 | 4.1198 |
| AMEF2 | 276 | 20711 | 4.1042 |
| LEF1TCF1 | 531 | 42880 | 3.9033 |
| ETS1 | 295 | 22501 | 3.8768 |

b) Transcription factor binding sites significantly enriched in the promoters of the 300 most downregulated genes

| **name** | **number of hits**  **in the submitted regions** | **total number of**  **hits on genome** | **log_10_(p-value)** |
| --- | --- | --- | --- |
| ETS1 | 333 | 22501 | 8.274 |
| E2F1DP2 | 449 | 34433 | 4.8228 |
| PEA3 | 294 | 21887 | 4.2225 |
| E2F1DP1 | 486 | 38173 | 4.2186 |

c) Transcription factor binding sites found specifically in the promoters of the top 300 upregulated genes (subtracted sites found in 3b)

| ATF | FOXO1 |
| --- | --- |
| ATF4 | XBP1 |
| NRF2 | RORA1 |
| MINI20 | ZTA |
| CREBATF | E4F1 |
| GLI | SMAD3 |
| NFE2 | SOX5 |
| MYC | T3R |
| ATF1 | SOX9 |
| ATF3 | IK3 |
| CREBP1CJUN | TFIIA |
| NFKAPPAB | ARP1 |
| CREBP1 | CEBP |
| TAXCREB | E2 |
| ERR1 | GATA3 |
| RBPJK | CHX10 |
| HIF1 | S8 |
| SP3 | TFIII |
| VBP | STAF |
| HES1 | HFH1 |
| DR3 | MEIS1 |
| IK1 | HP1SITEFACTOR |
| HNF4ALPHA | TCF4 |
| PITX2 | E47 |
| NMYC | BACH1 |
| AP4 | FOXO4 |
| ZNF219 | IRF1 |
| RFX1 | AREB6 |
| FOXP3 | EGR1 |
| CIZ | AMEF2 |
| POU1F1 | LEF1TCF1 |

d) Transcription factor binding sites found specifically in the promoters of the top 300 downregulated genes (subtracted sites found in 3a)

| PEA3 |
| --- |

Supplementary methods and Supplementary Figure III - further detail on parallel plate flow apparatus.


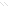


Cells

Peristaltic pump

Reservoir

Cotton wool

bung

For all flow experiments, media was made up omitting hydrocortisone and supplemented with 3.35% dextran (31390-Sigma) to raise the viscosity to 1.5cP, equivalent to plasma. HUVEC passage 2-4 were seeded at high density onto gelatin-coated slides and cultured for 48-72 hours before flow was commenced. Slides were placed in a parallel plate flow apparatus [1] and exposed to an oscillatory flow of 0±5 dynes/cm^2^ (OSS), a laminar shear stress of 15 dynes/cm^2^ (LSS15), or a laminar shear stress of 75 dynes/cm^2^ (LSS75) for 24 hours. For LSS15 or LSS75, the pump speed was increased slowly and maintained at ~5 dynes/cm^2^ for 20 minutes to allow the media to warm and equilibrate CO_2_, before being increased slowly to the final speed.


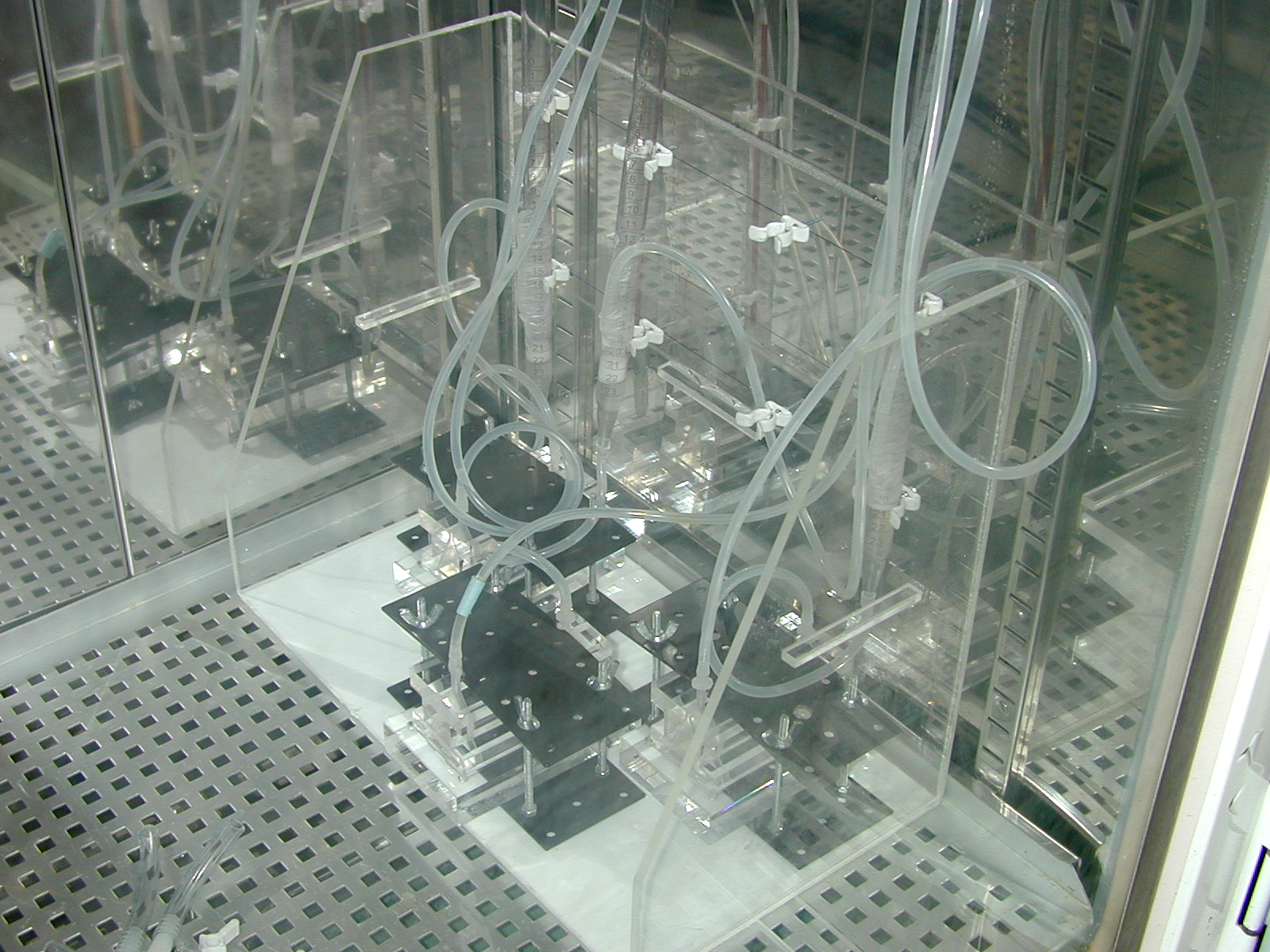


Supplementary Methods - *Gene expression analysis*

In brief, bead summary intensities were log2-transformed and then normalized using inter-array quantile normalization. Differential expression between the treatments of interest was assessed using a Baysian statistics moderated t-test [2]. Genes were considered significant if the P <0.05 after adjusting for multiple testing by using Benjamini and Hochberg's method [3]. The false discovery rate (FDR) was thereby controlled to be <5%. Whole genome rVISTA analysis was performed on the 300 most upregulated and downregulated genes to identify evolutionary conserved transcription factor binding sites enriched in the promoters of these gene sets (<http://genome-test.lbl.gov/cgi-bin/WGRVistaInputCommon.pl>) [4]. In a complementary approach Gene Set Enrichment Analysis [5] was used for analysis of pathway-based differential gene expression, and for enrichment of transcription factor binding sites in promoters of differentially expressed genes, using MySigdb build version 2.5.

Supplementary Table IV. primer sequenced used.

| **Primer Number** | **Gene Target** | **Sequence** |
| --- | --- | --- |
| SW321F | ARHGDIB | CCGGACAGAGACGTGAAGCA |
| SW322R | ARHGDIB | GACCATCTCCCAGCAGCGTT |
| SW394F | ASNS | GGCTGCCTTTTATCAGGGGG |
| SW395R | ASNS | GGGCTGTCTTCCATGCCAAT |
| SW379F | ATF2 | GGTCGTCGGAGAAGAGCAGC |
| SW380R | ATF2 | TGGCGGTTACAGGGCAATCT |
| SW381F | ATF-3 | CCCTCCTGGGTCACTGGTGT |
| SW431F | ATF4 | ACTTTGCCCGCCCACAGATGTA |
| SW432R | ATF4 | TCAAGTCCCCCACCAACACCTCGC |
| SW331F | C10orf58 | GCGGATCTGTCCTCCCTGAA |
| SW332R | C10orf58 | CCAGAGAAGCCTCCGTTCCA |
| SW317F | CTGF | GGTGTACCGCAGCGGAGAGT |
| SW318R | CTGF | GGGCCAAACGTGTCTTCCAG |
| SW345F | DUSP1 | CGGAGCTGTGCAGCAAACAG |
| SW346R | DUSP1 | CCTTGCGGGAAGCGTGATAC |
| SW347F | DUSP5 | AGTGCGAGTTCCTCGCCAAC |
| SW348R | DUSP5 | AAGCCATGCAGATGGTGGGT |
| SW439F | DUSP6 | GCCCGGGGCAAGAACTGTGGT |
| SW440R | DUSP6 | AGGCATCGTTCATCGACAGATTGAGC |
| SW349F | DUSP8 | GCCGCAGACAGCTTCCTCTC |
| SW350R | DUSP8 | GCGAGCCCAGGTAGAGGTGA |
| SW351F | DUSP16 | TCCTGCGTGTGCCTGTGAAT |
| SW352R | DUSP16 | TCCAGGAGTTGGCCCAGAAA |
| SW417F | DUSP23 | TGGACGAGGCCAACGCACGG |
| SW418R | DUSP23 | GCAGCCAAGCCCCGCTCCTT |
| SW319F | EDN1 | TGTTTGTGGCTTGCCAAGGA |
| SW320R | EDN1 | GGAACAACGTGCTCGGGAGT |
| SW204F | eNOS | GCCGGAACAGCACAAGAGT |
| SW205R | eNOS | GCGGCCTTGGCATCCTC |
| SW311F | FGF16 | CATCCGGGGAGTGGACTCTG |
| SW312R | FGF16 | CGGGGTGAGCCATCTTTGTT |
| SW323F | GIMAP1 | TCTGCACTCACAGCCGAAGG |
| SW324R | GIMAP1 | CTGTTCCCAGTGGCGCTCTT |
| SW325F | GIMAP4 | AGCACACCAGGGGCCAGTTA |
| SW326R | GIMAP4 | TCCATGAGCTGCTGCGTTTC |
| SW327F | GIMAP7 | GCAACAGCTCAAGCAGCCTC |
| SW328R | GIMAP7 | TGGTAACAGCTTGGGCAGCA |
| SW329F | GIMAP8 | CCCCTCACCCTCCTCACCTT |
| SW330R | GIMAP8 | TGCCCAGAATGGCATTTCCT |
| SW375F | GPX1 | CGAGAATGTGGCGTCCCTCT |
| SW376R | GPX1 | CTTGGCGTTCTCCTGATGCC |
| SW355F | HSPA1A | TGAGGAGCTGCTGCGACAGT |
| SW356R | HSPA1A | GGCTGGAAACGGAACACTGG |
| SW357F | HSPA1B | TGTTGAGTTTCCGGCGTTCC |
| SW358R | HSPA1B | AACACCCCCACGCAGGAGTA |
| SW359F | HSPA5 | GTGCTCCTGTGCTACGGCCT |
| SW360R | HSPA5 | CCGACGCAGGAGTAGGTGGT |
| SW353F | HSPA6 | TGAAGCCGAGCAGTACAAGGC |
| SW354R | HSPA6 | TGTCTTGCATTTTGCGCCTG |
| SW276F | hTIMP1 | GATACTTCCACAGGTCCCACAACC |
| SW277R | hTIMP1 | CAGCCAACAGTGTAGGTCTTGGTG |
| SW278F | hTIMP2 | GAAGGAAGTGGACTCTGGAAACGA |
| SW279R | hTIMP2 | ATGAAGTCACAGAGGGTGATGTGC |
| SW280F | hTIMP3 | CTTCCGAGAGTCTCTGTGGCCTTA |
| SW281R | hTIMP3 | CTCGTTCTTGGAAGTCACAAAGCA |
| SW282F | hTIMP4 | AAGCCAACAGCCAGAAGCAGTATC |
| SW283R | hTIMP4 | CAGGGTACTGTGTAGCAGGTGGTG |
| SW413F | IL-6 | TGTGAAAGCAGCAAAGAGGCACTGGC |
| SW414R | IL-6 | TCTGCACAGCTCTGGCTTGTTCCTCAC |
| SW313F | IL8 | TGGCTCTCTTGGCAGCCTTC |
| SW314R | IL8 | CCCAGTTTTCCTTGGGGTCC |
| SW309F | ITGB8 | CACCCTCACAATTTGTCTCAGGC |
| SW310R | ITGB8 | GGCTGACACTCTGTAATCTGATGAGG |
| SW218F | KLF2 | GTGAGAAGCCCTACCACTGCAACT |
| SW219R | KLF2 | CCGGTTCTCTGGGTCCAATAAATA |
| SW369F | KLF4 | TGGACCCCCTCTCAGCAATG |
| SW370R | KLF4 | CTCTTGGTAATGGAGCGGCG |
| SW421F | KLF6 | TGCTCATGGGAAGGGTGTGA |
| SW422R | KLF6 | CATGCTTTGGCTGGAACACG |
| SW423F | MT1A | CACGTGCGCCTTATAGCCTCTC |
| SW424R | MT1A | GTCAGGGTTGTATGGAAAAAAAATTCC |
| SW425F | MT1E | GCCCGGCCCGACCTCCGTCT |
| SW426R | MT1E | CGGCGCACGTGCAGGAGCCA |
| SW427F | MT1F | GCAGCGGCCGGCTGTTGGGG |
| SW428R | MT1F | GCCCTGGGCACACTTGCTACAGCC |
| SW299F | MT1G | CCACGTGCACCCACTGCCTCTTCC |
| SW300R | MT1G | AGTACAAATAGAGTGACCCGTAAAAT |
| SW297F | MT1M | GTCGCTCCATTTATCGCTTGAG |
| SW298R | MT1M | CAGCAAATGGCTCAGTATCGTATTG |
| SW295F | MT1X | TCTGTCCCGCTGCGTGTTTTC |
| SW296R | MT1X | CCAGGACAGCTGTGCTCTCAGATGT |
| SW429F | MT2A | TGCCGCGCTGCACTCCACCA |
| SW430R | MT2A | GCAGCCCTGGGCACACTTGGCA |
| SW263F | NOX1 | aaaccacctcttgacaatgggaaa |
| SW264R | NOX1 | gaaggcatccacaaacaggaaaac |
| SW265F | NOX2 | atctacctcactggctgggatgag |
| SW266R | NOX2 | gggtgttgacttgcaattgtcttg |
| SW222F | NOX4 | atatccggagcaataagccagtca |
| SW223R | NOX4 | gattgaatgaagggcagaatttcg |
| SW269F | NOX5 | TGTATCTGAACATCCCCACCATTG |
| SW270R | NOX5 | TCCTCGACAGCCTCTTAGAACCAC |
| SW254F | p47phox | cgatcaatccagagaacaggatca |
| SW255R | p47phox | gtactcttgccatctttgggcatc |
| SW392F | P67phox | CATTGGCCACGAGCATGAAG |
| SW393R | P67phox | ATCCACCACAGATGCCACGA |
| SW337F | PLAT | CCTGCCTGCTCTGAGGGAAA |
| SW338R | PLAT | TATGTTTGCCCAGGCCCAGT |
| SW339F | PLAUR | AGGACCTCTGCAGGACCACG |
| SW340R | PLAUR | TCGGGAATAGGTGACAGCCC |
| SW388F | RNF115/ZNF364 | CAGAGCTTTGGGGCCATTTG |
| SW389R | RNF115/ZNF364 | GCAACCGTGGAGGTCTTGCT |
| SW307F | S100P | CCCAGGAGGAAGGTGGGTCT |
| SW308R | S100P | TCCACCTGGGCATCTCCATT |
| SW305F | SLC30A1 | ACGCCATCTTCCTGACTGGC |
| SW306R | SLC30A1 | CTCTTAACGCGAGGCCCCTT |
| SW371F | SMAD7 | GTGTTCAGGTGGCCGGATCT |
| SW372R | SMAD7 | CGGAGGAAGGCACAGCATCT |
| SW361F | SNRK | CAGCAACAAATTTCAACCAGGG |
| SW362R | SNRK | TGCCCACACACCAACATGAA |
| SW411F | Stat-1 | GCCACCATCCGTTTTCATGACC |
| SW412R | Stat-1 | TGCTCTGAATATTCCCCGACTGAG |
| SW373F | THBD | CAACACACAGGGTGGCTTCG |
| SW374R | THBD | GGCTGGACAGGCAGTCTGGT |
| SW315F | THBS1 | CATCAATGGAGGCTGGGGTC |
| SW316R | THBS1 | CAGGGATTGGACAGGCATCC |
| SW232F | VCAM-1 | GAACCCAAACAAAGGCAGAGTACG |
| SW233R | VCAM-1 | TGCTTCTTCCAGCCTGGTTAATTC |
| SW398F | VIP | GCCAGGCATGCTGATGGAGTTTTCACC |
| SW399R | VIP | ACTGCATCTGAGTGACGTTTGACTGGT |
| SW433F | XBP1 | CGCAGCACTCAGACTACGTGCACCTCT |
| SW434R | XBP1 | CCAGGCTGGCAGGCTCTGGGG |

References for supplementary information:

[1] Castier Y, Ramkhelawon B, Riou S, Tedgui A, Lehoux S. Role of NF-kappa B in Flow-Induced Vascular Remodeling. Antioxidants & Redox Signaling 2009; 11:1641-1649.

[2] Baldi P, Long AD. A Bayesian framework for the analysis of microarray expression data: regularized t-test and statistical inferences of gene changes. Bioinformatics 2001; 17:509-519.

[3] Benjamini Y, Hochberg Y. Controlling the False Discovery Rate - a Practical and Powerful Approach to Multiple Testing. J R Stat Soc Ser B-Methodol 1995; 57:289-300.

[4] Zambon AC, Zhang L, Minovitsky S, Kanter JR, Prabhakar S, Salomonis N, Vranizan K, Dubchak I, Conklin BR, Insel PA. Gene expression patterns define key transcriptional events in cell-cycle regulation by cAMP and protein kinase A. Proc Natl Acad Sci U S A 2005; 102:8561-8566.

[5] Subramanian A, Tamayo P, Mootha VK, Mukherjee S, Ebert BL, Gillette MA, Paulovich A, Pomeroy SL, Golub TR, Lander ES, Mesirov JP. Gene set enrichment analysis: A knowledge-based approach for interpreting genome-wide expression profiles. Proc Natl Acad Sci U S A 2005; 102:15545-15550.
